# A four-column theory for the origin of the genetic code: tracing the evolutionary pathways that gave rise to an optimized code

**DOI:** 10.1186/1745-6150-4-16

**Published:** 2009-04-24

**Authors:** Paul G Higgs

**Affiliations:** 1 Dept of Physics and Astronomy, McMaster University, Hamilton, Ontario L8S 4M1, Canada

## Abstract

**Background:**

The arrangement of the amino acids in the genetic code is such that neighbouring codons are assigned to amino acids with similar physical properties. Hence, the effects of translational error are minimized with respect to randomly reshuffled codes. Further inspection reveals that it is amino acids in the same column of the code (i.e. same second base) that are similar, whereas those in the same row show no particular similarity. We propose a 'four-column' theory for the origin of the code that explains how the action of selection during the build-up of the code leads to a final code that has the observed properties.

**Results:**

The theory makes the following propositions. (i) The earliest amino acids in the code were those that are easiest to synthesize non-biologically, namely Gly, Ala, Asp, Glu and Val. (ii) These amino acids are assigned to codons with G at first position. Therefore the first code may have used only these codons. (iii) The code rapidly developed into a four-column code where all codons in the same column coded for the same amino acid: NUN = Val, NCN = Ala, NAN = Asp and/or Glu, and NGN = Gly. (iv) Later amino acids were added sequentially to the code by a process of subdivision of codon blocks in which a subset of the codons assigned to an early amino acid were reassigned to a later amino acid. (v) Later amino acids were added into positions formerly occupied by amino acids with similar properties because this can occur with minimal disruption to the proteins already encoded by the earlier code. As a result, the properties of the amino acids in the final code retain a four-column pattern that is a relic of the earliest stages of code evolution.

**Conclusion:**

The driving force during this process is not the minimization of translational error, but positive selection for the increased diversity and functionality of the proteins that can be made with a larger amino acid alphabet. Nevertheless, the code that results is one in which translational error is minimized. We define a cost function with which we can compare the fitness of codes with varying numbers of amino acids, and a barrier function, which measures the change in cost immediately after addition of a new amino acid. We show that the barrier is positive if an amino acid is added into a column with dissimilar properties, but negative if an amino acid is added into a column with similar physical properties. Thus, natural selection favours the assignment of amino acids to the positions that they occupy in the final code.

**Reviewers:**

This article was reviewed by David Ardell, Eugene Koonin and Stephen Freeland (nominated by Laurence Hurst)

## Background

It is well known that the arrangement of amino acids in the standard genetic code is distinctly non-random and is such that neighbouring codons (i.e. those that differ at only one of the three positions) are assigned to amino acids with similar physical properties. Given that naturally occurring protein sequences have already been selected for efficient function for long periods, most random amino acid changes introduced into a protein sequence are likely to be deleterious. The magnitude of the deleterious effect is likely to be larger for amino acid substitutions that make a larger change in the physical properties. A mutation at a single DNA site will cause an amino acid to be replaced by the amino acid assigned to the neighbouring codon; hence the arrangement of genetic code is such that mutations are likely to be less deleterious than in a random code. Errors in proteins also arise during translation, i*.*e. a mispairing between the tRNA and the mRNA introduces an amino acid into the protein that is different from the one specified by the gene sequence. The most frequent mispairing events are likely to involve only one out of three of the nucleotide pairs between the codon and anticodon; hence a translational error has a similar effect to a point mutation. It thus appears that the standard code is optimized to reduce the effects of both translational error and deleterious mutations.

These conclusions are reached by studies that compare the standard code with large numbers of randomly reshuffled codes [1-8]. It is usually assumed that translational error is more important than deleterious mutations. A function Φ is defined that measures the mean cost of a translational error. This depends on the arrangement of amino acids in the code. Φ is calculated for the real code and for many random codes. The fraction of random codes, *f*, that have lower cost than the real code is then obtained. The standard code is often said to be 'one in a million', because *f *= 10^-6 ^in the study of Freeland and Hurst [2]. The value of *f *varies widely among the studies cited above and it depends on many details of the way the cost function is defined. For our purposes, the precise value of *f *is unimportant. We accept that *f *is small for reasonable choices of the cost function and we focus on the question of how this came to be. The obvious implication is that evolution and natural selection have shaped the code in some way.

The majority of bacteria, archaea and eukaryotic nuclear genomes use the standard code that has remained unchanged since the common ancestor of these three domains of life. However, many alternative codes exist in modern organisms in which one or more codons have been reassigned from their meaning in the standard code [9-11]. These alternative codes demonstrate that evolution of the code is possible and that it is not completely frozen. A shortcoming of statistical studies on randomly reshuffled codes is that they do not provide an evolutionary mechanism by which an optimized code is reached. Clearly, evolution does not proceed by creating a million random alternatives and selecting the best. We need to understand how a code can change by gradual steps, and why each successive code was selected over the previous one. Dynamical models have also been studied [12,13] in which the code changes by swapping the amino acids assigned to two codon blocks. If the swap leads to a decrease in Φ, the change is accepted. This model shows that the code can evolve by a series of local moves towards a local optimum. The local optima typically have much smaller Φ than random codes; hence *f *is small for the local optima codes that are reached by the evolutionary trajectories. Both these papers concluded that the standard code is not a local optimum because there are some swaps that can reduce Φ further. However, the standard code is still much better than most random codes, and hence has a small *f*.

Whether the real code is a local optimum depends on the set of local moves allowed. We believe that allowing swaps of the positions of any two amino acids is unrealistic, and overestimates the ease with which the code can change. None of the many known examples of codon reassignment in alternative codes occurs by swapping the amino acids assigned to two codon blocks [11]. Instead, one or more codons assigned to one amino acid are reassigned to another, so that one block of codons decreases in size and the other increases. Furthermore, the amino acid that acquires the codon is almost always a neighbour of the one that loses it (e*.*g. the AUA codon, which is initially Ile, can be reassigned to Met, which initially occupies the neighbouring AUG codon). The reason for this is that reassignments of codons to neighbouring amino acids can be done by changing only a single base in the tRNA anticodon (see examples in [11]). The local moves used in [12] and [13] do not incorporate these constraints. Secondly, this picture assumes that all 20 amino acids are present initially, whereas it seems more likely that the code began with a small number of amino acids and built up gradually by sequential addition of new amino acids. In a code that contains 20 amino acids, the factor on which selection can operate is proportional to the translational error rate. If the error rate were zero, then the cost function Φ would be zero, and all codes would be equivalent. On the other hand, if the code evolves by sequential addition, then there is a major selective advantage every time a new amino acid is added because vastly more proteins can be made with an alphabet of *n*+1 amino acids than with an alphabet of *n*. This advantage is not dependent on the error rate and still exists even if there is no translational error, as we will show below. A key step in the theory we present in this paper is to modify the cost function to deal with codes with fewer than 20 amino acids. Although translational error does play a role in the theory below, it is positive selection for increasing protein diversity that drives the evolution of the code, rather than error minimization. We will show that the dynamics of addition of new amino acids leads to a code in which error rates are minimized (i*.*e*. f *is small), even though this is not the driving force.

Following Crick [14], the genetic code is sometimes described as a frozen accident. It is clear that codon reassignments have occurred in relatively recent evolutionary history because there are many different changes in separate lineages of mitochondria, so the code is not strictly frozen. Nevertheless, the conclusion we draw from our previous studies of codon reassignment [10,11] is that it is rather difficult to change the code after a full code with 20 amino acids has become established. Codon reassignment requires either that the codon disappears completely from the genome during the reassignment process, or that the population passes through a deleterious intermediate stage when a codon is either ambiguous or unassigned. All these possibilities are unlikely events, but they become slightly easier in small genomes where the number of occurrences of any given codon is small. This explains why the majority of codon reassignments occur in the small genomes of mitochondria. There are virtually no codon reassignments in prokaryotes or in most groups of eukaryotes, and there are a large number of codons that have never been found to be reassigned in genomes of any type. As reshuffling the positions of amino acids in the code at a late stage of evolution is difficult, the easiest way to ensure that an amino acid ends up in a good position in the code is to assign it to a good position in the first place. In other words, we will argue that the selective forces that lead to the non-random positioning of amino acids in the code act at the time when a new amino acid is first added, rather than during subsequent reshuffling events. The strongest non-random pattern in the standard code is that amino acids in the same column have similar physical properties, which has been noted by many authors [15-18,3]. This pattern is not an accident, because the dynamics of natural selection drive the addition of new amino acids into positions that are consistent with this pattern, as we will show below. However, we will argue that this pattern is indeed frozen, because it has remained in place since the earliest stages of code evolution.

This theory owes a debt to the coevolution theory for the origin of the genetic code developed by Wong [19,20] and Di Giulio [21,22], and shares several important features with it. The coevolution theory proposes that the first code incorporated only a small number of amino acids and that the later amino acids were added sequentially to the code. The earliest amino acids are those that are simplest to synthesize, either by prebiotic chemistry in the environment or by short metabolic pathways inside the cell. The later amino acids are those that cannot be synthesized non-biologically, and require multi-step metabolic pathways to synthesize inside the cell. We have recently carried out our own survey of amino acid frequencies in non-biological contexts [23,24]. This leads to a prediction of which amino acids are early and late that is largely in agreement with that proposed in the coevolution theory. The coevolution theory also supposes that early amino acids initially were assigned to large codon blocks, and that as new amino acids were added, the earlier amino acids relinquished some of their codons to the newer ones. We will refer to this as 'subdivision', meaning that a large codon block is divided into two, one of which is retained by the earlier amino acid and one of which is reassigned to the new amino acid. The code also evolves by subdivision according to our theory. Another essential aspect of the coevolution theory is that the positions in which the later amino acids are added are determined by the precursor-product relationships. A product amino acid takes over some of the codons of its precursor. In our theory, this is usually not true. We will consider this aspect of the theory below, and we will show that natural selection does not usually favour the addition of an amino acid in a position that follows its precursor-product relationship; instead the favourable positions for addition of an amino acid are determined by its physical properties.

This theory also owes a debt to the models for code evolution of Ardell and Sella [25-28]. These models emphasize the role of code-message coevolution. If a new variant code appears in an organism, it has to translate the genes that are already present. These genes are adapted to the previous code. If the new code variant leads to an increase in fitness of proteins translated from these genes, then the new code can spread. After the code spreads, a further increase in fitness is possible when the gene sequences adjust to the new code. We will use this same idea below. In Ardell and Sella's models, the code is presumed to begin in an ambiguous state where codons are assigned to groups of amino acids. This code would produce ensembles of proteins rather than well specified sequences. Code evolution progresses towards a well-defined state where every codon is assigned to only one amino acid. It is shown that the final state tends to be one in which error is minimized. Another model that begins with ambiguous codons is that of Vetsigian *et al*. [29]. This model emphasizes the role of horizontal gene transfer during the evolution of the code. Organisms that share the same code can also share transferred genes. Therefore there is an advantage to using common codes. It could be true that horizontal transfer was frequent at the time of the origin of the code and, if so, this could help to explain why a universal code spread through all organisms. However, a new and fitter code could also spread by vertical descent; therefore, for the theory that follows, it is unimportant whether there was a high rate of horizontal transfer.

The models in [25-29] are somewhat abstract, in that they do not consider specific amino acids or try to predict the specific layout of the real code. In contrast, our aim here is to try to explain why particular amino acids end up in particular places in the real code. For this reason, we need a realistic measure of the cost of substitution of one amino acid by another. In this paper, we will proceed as follows. Firstly, we will consider the choice of matrix of amino acid substitution costs. Then we will develop a measure for the cost of the code, which is based on the standard Φ function used in studies of randomly reshuffled codes, but which applies to codes with variable numbers of amino acids. From this, we can consider the dynamics of addition of a new amino acid by the subdivision process, and predict which amino acids should be selected to be added into which positions of the code. We propose that the code had a four-column layout early on, and show that the properties of the current code can be understood as a natural outcome of the dynamics of code evolution beginning from this state.

### Choice of a cost function for amino acid substitutions

A basic ingredient of the Φ function that measures the cost of a code is the function *g*(*a, b*), which is the cost of insertion of amino acid *b *into a site where amino acid *a *is preferred. The cost is presumed to derive from the reduced level of functionality of the protein sequence if a non-optimal amino acid is used or from the toxicity of misfolded proteins that might arise when translational errors are made. The cost function is presumed to be symmetrical, i*.*e*. g*(*a, b*) = *g*(*b, a*), and such that *g*(*a, a*) = 0. The cost is larger for amino acids with more dissimilar physical properties. It may therefore be thought of as a distance between amino acids in physical property space.

Most previous studies have considered cost functions that depend on differences between single physical properties. In particular, the difference in the polar requirement (PR) scale [30] is often used (this is listed in column 9 of Table 1). Studies of reshuffled codes have shown that the code appears to be more optimized with respect to PR than any other single property [1]; therefore it is clear that PR is a relevant property. However, we do not think that it is possible to adequately represent molecular properties by a single number. Higgs and Attwood [31] defined a distance based on 8 properties from the protein folding literature (listed in columns 1–8 of Table 1, and see [32-37]). These properties were normalized such that the mean was 0 and the standard deviation was 1, for each property. Let *z*_*ka *_be the normalized value of property *k *for amino acid *a*. A useful distance measure is the Euclidean distance between amino acids in the 8-dimensional *z *space. These properties are correlated with each other to a considerable extent. A principal component analysis shows that 79% of the variance of these 8 properties is explained by the first two component axes. The projection of the amino acids onto the first two components is shown in Chapter 2 of [31] and in [18]. This makes it clear that amino acids in the first and second columns of the code form two tight clusters in property space. Those in the third column form a rather more disperse cluster, while those in the fourth column are not clustered at all. The fourth column contains the smallest (Gly), largest (Trp) and most basic (Arg) amino acids, as well as Cys, which is unusual due to the formation of disulphide bonds. The principal components plot also shows that there is no particular similarity between amino acids in the same row. The small value of *f *seen in random code studies therefore derives from the similarity of amino acids in columns and not rows. Urbina *et al*. [18] showed that several aspects of protein sequence evolution can be understood from this distance matrix. Substitution rates at 1^st ^position are faster than those at 2^nd ^position because 1^st^-position substitution rates are more conservative than 2^nd^-position substitutions. Also, the frequencies of amino acids vary among genomes because of the variation in base frequencies that arises from biased mutation rates. It was shown that amino acids in the 1^st ^and 2^nd ^columns of the code vary substantially under mutation pressure, whereas those in the 3^rd ^and 4^th ^columns vary much less. This can be predicted based on the degree of similarity of an amino acid to its neighbouring amino acids in the code.

**Table 1:** Amino acid physical properties.

Properties:	1	2	3	4	5	6	7	8	9	Frequency (%)
F – Phe	135	19.80	0.35	5.48	2.8	3.7	218	0.88	5.0	4.39
L – Leu	124	21.40	0.13	5.98	3.8	2.8	180	0.85	4.9	10.15
I – Ile	124	21.40	0.13	6.02	4.5	3.1	182	0.88	4.9	6.95
M – Met	124	16.25	1.43	5.74	1.9	3.4	204	0.85	5.3	2.28
V – Val	105	21.57	0.13	5.96	4.2	2.6	160	0.86	5.6	7.01
S – Ser	73	9.47	1.67	5.68	-0.8	0.6	122	0.66	7.5	6.46
P – Pro	90	17.43	1.58	6.30	-1.6	-0.2	143	0.64	6.6	4.26
T – Thr	93	15.77	1.66	6.16	-0.7	1.2	146	0.70	6.6	5.12
A – Ala	67	11.50	0.00	6.00	1.8	1.6	113	0.74	7.0	7.80
Y – Tyr	141	18.03	1.61	5.66	-1.3	-0.7	229	0.76	5.4	3.30
H – His	118	13.69	51.60	7.59	-3.2	-3.0	194	0.78	8.4	2.03
Q – Gln	114	14.45	3.53	5.65	-3.5	-4.1	189	0.62	8.6	3.45
N – Asn	96	12.28	3.38	5.41	-3.5	-4.8	158	0.63	10.0	4.37
K – Lys	135	15.71	49.50	9.74	-3.9	-8.8	211	0.52	10.1	6.32
D – Asp	91	11.68	49.70	2.77	-3.5	-9.2	151	0.62	13.0	5.19
E – Glu	109	13.57	49.90	3.22	-3.5	-8.2	183	0.62	12.5	6.72
C – Cys	86	13.46	1.48	5.07	2.5	2.0	140	0.91	4.8	1.10
W – Trp	163	21.67	2.10	5.89	-0.9	1.9	259	0.85	5.2	1.09
R – Arg	148	14.28	52.00	10.76	-4.5	-12.3	241	0.64	9.1	5.23
G – Gly	48	3.40	0.00	5.97	-0.4	1.0	85	0.72	7.9	6.77

Weights:	0.000	0.155	0.000	0.028	0.218	0.000	0.277	0.179	0.142	-----

Definitions of the 9 properties used to construct the distance matrix:

1. Volume from van der Waals radii [32].

2. Bulkiness – a measure of the shape of the side chain [33].

3. Polarity – a measure of the electric field strength around the molecule [33].

4. Isoelectric point [33].

5. Hydrophobicity scale [34].

6. Hydrophobicity scale [35].

7. Surface area accessible to water in an unfolded peptide [36].

8. Fraction of accessible area lost when a protein folds [37].

9. Polar requirement [30].

The frequencies column shows the mean percentage of each amino acid in the protein sequences of modern organisms [6].

Regrettably, PR was not included in our original choice of 8 properties because it does not appear in the protein folding literature, even though it is prominent in the genetic code literature. Recently, we have added PR as a ninth property. We have also considered weighted distance measures, *d*_*W*_, that allow different weights to be assigned to different properties:

(1)

where the *z*_*ka *_are the normalized property values and *w*_*k *_is the weight assigned to property *k*. In this paper, we set *g*(*a, b*) = *d*_*W*_(*a, b*). The initial multiplying constant makes no difference for the conclusions of this paper; therefore we multiply by a constant such that the mean distance between pairs of non-identical amino acids is 100. The values of the weights in equation 1 were estimated by fitting an evolutionary model to sequence data using a maximum likelihood method [38]. A codon-based substitution model was used in which the rate of substitution from codon *i *to codon *j *is defined as

(2)

where *α*_*cat *_is a rate parameter that depends on whether there are 1, 2 or 3 substitutions between codons, *κ*_*ij *_is a factor that distinguishes transitions and transversions, and *π*_*j *_is the equilibrium frequency of codon *j*. The amino acids assigned to codons *i *and *j *are *a*_*i *_and *a*_*j*_. The model supposes that the rate of non-synonymous substitutions is an exponentially decreasing function of the distance between the amino acids. *D *is a parameter that controls the rate of this decrease. Details of the model and the procedure for estimating the parameters are explained in [38]. Having chosen the maximum likelihood values of the weights, the distance *d*_*W *_should be a realistic measure of the distance between amino acids that is actually 'seen' by evolution.

Previous authors have tried to use PAM substitution matrices to define cost functions for use in genetic code studies [4,8]. However, this is known to be problematic because the PAM scores depend on the structure of the code (i*.*e. on how many DNA substitutions are required to change the amino acid); therefore using PAM scores to measure optimality of the code is circular. Our method avoids this problem because the rate defined in equation 2 separates the factors that depend on mutation rate and number of substitutions (*α*_*cat*_*κ*_*ij*_*π*_*j*_) from the factor that depends on amino acid properties (exp(-*d*_*W*_/*D*)). Thus, the *d*_*W *_matrix that we obtain can be used as a cost function with no problem of circularity.

The largest data set that we have analyzed with this model is a set of paralogous gene pairs from *S. cerevisiae*. The weight parameters obtained from this are shown at the bottom of Table 1 (specifically, these are the values from model W3, shown in Table Four of [38]). In this paper we will use these values of the weights to define *d*_*W*_. Waglechner [39] considered several other data sets and showed that the weights are surprisingly consistent between sequences from different organisms. There are three properties for which *w*_*k *_= 0, i*.*e. the distance does not depend on these properties. This is due to the relatively strong correlation between some of the properties. If one property captures an aspect of the data well, the weight associated with a slightly different, but strongly correlated property can become zero.

**Figure 1:**
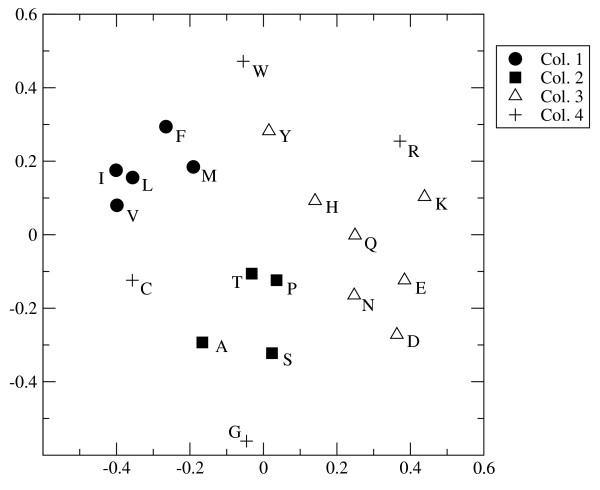
**Two-dimensional visualization of the matrix of physical property distances, *d*_*W*_, obtained via multi-dimensional scaling**. The clustering of the amino acids in the first three columns of the genetic code is apparent. The horizontal axis is related to hydrophobicity and the vertical axis is related to size of the amino acid.

The choice of distances is important in what follows; therefore the full *d*_*W *_matrix with these weight values is shown in Additional file 1. As a help to visualize what these distances mean, Figure 1 shows a two-dimensional representation of the matrix obtained by multi-dimensional scaling using the Permap program [40]. The program positions points in two dimensions such that the distances in the two-dimensional space are as close as possible to the original distances in the multi-dimensional space. The result is invariant to rotation; therefore we have rotated the figure so that the similarity with the principal components plot of [31] and [18] can be seen. The *x *axis correlates strongly with hydrophobicity and the *y *axis correlates strongly with size. Figure 1 also illustrates that the amino acids in the first three columns of the genetic code are clustered in property space, but those in the fourth column are not. It also shows several other points that are intuitively reasonable, *e.g*. the particularly tight clustering of the three amino acids with simple hydrocarbon side chains (I, L and V), and the closeness of the two acids (D and E), the two amines (N and Q), the two most basic amino acids (R and K), and the aromatic amino acids (F, Y and W).

In summary, the weighted distance measure defined in this way is our best estimate of an evolutionarily meaningful distance measure, and we will use this as a cost function in this paper. Note that the multidimensional distance defined in equation 1 is used. The projection to two dimensions in Figure 1 is merely shown as a visual aid.

### Defining cost and fitness for codes with fewer than 20 amino acids

We begin with the standard function for the cost of the code used for comparing alternative codes with 20 amino acids [6-8,13]. For any given substitution cost function, *g*, the mean cost of an error, Φ, is defined to be the average value of *g *for all possible types of error, weighted by the frequency with which they occur. This can be written

(3)

where *F*_*i *_is the frequency of codon *i *in the gene sequences, *p*_*ij *_is the probability that codon *i *is mistranslated as codon *j *and *a*_*i *_and *a*_*j *_are the amino acids assigned to codons *i *and *j*. Φ is a property of the arrangement of amino acids in the code. Thus, for convenience, we will refer to Φ as the 'cost of a code'. We will follow Gilis *et al*. [6] and take

(4)

where *P*(*a*_*i*_) is the frequency of amino acid *a*_*i *_in the protein sequences of the organism and *n*(*a*_*i*_) is the number of codons assigned to *a*_*i *_in the code in question. This assumes that all codons for a given amino acid have equal frequency, but amino acids have different frequencies that can be estimated from protein sequence data. We will use the average amino acid frequencies calculated from representative genomes of all three domains of life [6]. These frequencies are reproduced in the final column of Table 1.

Let *ε *be a parameter that controls the rate of translational error. Following Freeland & Hurst [2], we will use a form of the error probability matrix that makes transition errors more frequent than transversion errors and makes 2^nd ^position errors less frequent than 1^st ^and 3^rd ^position errors.

(5)

if *i *and *j *differ by any change at third position or by a transition at first position;

0.5*ε *if *i *and *j *differ by a transversion at first position or a transition at second position;

0.1*ε *if *i *and *j *differ by a transversion at second position;

0 if *i *and *j *differ at more than one position.

The probability of correct translation is . With these choices of *F*_*i *_and *p*_*ij*_, Φ is equivalent to equation (2) of Gilis *et al*. [6]. Possible alternatives would be to assume all codons have equal frequencies (*F*_*i *_= 1/61 for all sense codons) or to assume that all single-base errors occur with equal probability (*p*_*ij *_= *ε *in each case). In fact, the evidence to support the relative rates of error in equation (5) is rather weak (see the comments of Ardell on the paper of Novozhilov *et al*. [13]). However, it is very likely true that the error rate at 2^nd ^position is lower than that at 1^st ^and 3^rd ^positions; therefore, we feel that the parameterization above is significantly better than setting all single-base errors rates to be equal.

For an evolutionary argument, we need to relate the cost, Φ, to fitness. Consider an organism that makes many proteins using a given code. At each site there is some probability of an error, and the cost of an error will be given by the *g *function for the substitution that occurs. If we assume that the costs of the errors are additive over sites, that there are many sites, and that each gene is translated many times, then the typical cost to an organism using this code is proportional the mean value of the *g *function, which is Φ, defined as in equation 3. If we then suppose that fitness varies linearly with cost, the typical fitness of an organism can be written *w *= 1 - *s*Φ, where *s *is a parameter representing the strength of selection against translational error. Thus, minimizing Φ is equivalent to maximizing fitness. This assumption is made implicitly by previous authors, although it is not often stated. We will also make the same assumption, but we wish to emphasize that it is only true if we assume that the costs and the fitness effects of errors are additive over sites.

Previous studies have assumed that the same set of 20 biological amino acids was present in all the random codes considered. We now wish to modify the Φ function to consider code variants with fewer than 20 amino acids that may have existed prior to the standard code. Suppose that, at each site in a protein, one particular amino acid is preferred according to selection for molecular structure and function. A site where amino acid *a *is preferred will be called a type-*a *site. We suppose the that the average frequencies of sites can be estimated from the frequencies of amino acids in current proteins, i*.*e. the frequency of type-*a *sites is *P*(*a*), as was used in equation 4. If the full set of amino acids is represented in the genetic code, then a codon for amino acid *a *will be used at every type-*a *site in the genome. The possible occurrence of codons for other amino acids at a type-*a *site is neglected, because it is assumed that translational error is more important than deleterious mutations.

Now consider a code with *K *amino acids, where *K *< 20. It is not always possible to use a codon for the preferred amino acid at every site because the preferred amino acid may not be in the code. For every amino acid *a*, let *B*(*a*) be the best available amino acid, i*.*e*. B*(*a*) is the amino acid in the current code for which *g*(*a*,*B*(*a*)) is smallest. If *a *is included in the code already, then *B*(*a*) = *a*, and *g*(*a*,*B*(*a*)) = 0. If *a *is not yet included in the code, then *B*(*a*) ≠ *a*, and *g*(*a*,*B*(*a*)) > 0. The best protein sequences that can be produced using the current code will use a codon for *B*(*a*) in every type-*a *site. We may now write

(6)

where *α *labels the type of site (*α *runs over all the 20 biological amino acids), and *φ*_*i*_(*α*) is the frequency of codon *i *at sites of type *α*. Suppose that the genome uses a codon for the best available amino acid at every site, and that synonymous codons are used with equal frequency. In this case

(7)

where the *δ*-function is 1 if the two arguments are equal and is 0 otherwise. This says that codon *i *will be used at sites of type *α *if the amino acid assigned to codon *i *is *B*(*α*). For a code with *K *= 20, *B*(*α*) = *α *for every *α*. In this case

(8)

If this is substituted into equation (6), we get back to equation (3), i*.*e. the modified Φ function is the same as the original Φ function for codes in which all amino acids are included. For codes with *K *< 20, it is not the same. Note that, if the error rate *ε *is a small parameter, the probabilities *p*_*ij *_are of order *ε *if *i ≠ j*, whereas the diagonal elements *p*_*ii *_are of order 1. As *g*(*a*_*i*_, *a*_*i*_) = 0, *p*_*ii *_does not contribute to Φ as defined in equation (3). Thus Φ is O(*ε*) for codes with *K *= 20, and Φ tends to zero if *ε *tends to zero. This is no longer true if *K *< 20. If *ε *tends to zero, then *p*_*ij *_= *δ *(*i, j*). Hence, from equation (6), Φ tends to a limiting value Φ_0_, given by

(9)

Φ_0 _is no longer a cost of translational error, it is a cost of using the best available amino acid instead of the preferred amino acid. As we are assuming fitness decreases linearly with cost, Φ_0 _should be interpreted as the reduction in the fitness of proteins using the best available amino acids with respect to 'ideal' proteins that use the preferred amino acids at every site. We now have a way of comparing codes with different numbers of amino acids, and we have a way of quantifying the advantage to introducing a new amino acid into the code.

The driving force for adding new amino acids is the reduction in Φ_0_, not the reduction in translational error. Φ_0 _will always decrease every time a new amino acid is added, because for every *α*, the best available amino acid after addition of a new code is either more similar to *α *than it was before or the same as it was before (i.e.* g*(*α,B*(*α*)) either decreases or stays the same). When the translational error rate, *ε*, is non-zero, the cost function in equation (6) contains the leading term, Φ_0_, and an O(*ε*) term that represents translational error. Although Φ_0 _decreases when a new amino acid is added, the O(*ε*) term usually increases because there are fewer synonymous substitutions when codons are divided into smaller blocks. The balance of the two terms therefore determines whether the addition of the new amino acid is favourable.

### Changes in the code cost function when a new amino acid is added

Suppose that the current code at any stage of evolution is defined by a specific set of assignments  of codons to amino acids, and that the current set of best available amino acids is *B*^*cur*^(*α*). Hence the codon frequencies at each type of site and the code cost can be determined:

(10)

(11)

Now suppose a change happens in the code such that a former block of codons is subdivided, and a new amino acid is added to the code. The new code is defined by . For the codons that have been reassigned,  is an amino acid that was not previously in the code. For the majority of codons that have not changed assignment, . Immediately after this change in the translation system, the gene sequences are the same as they were before; therefore the codon frequencies are still the same as before. In this intermediate state the code cost is

(12)

where the amino acids of the new code appear in the cost function but not in the codon frequencies. After the change has occurred, the new set of best available amino acids is *B*^*new*^(*α*). Mutations can occur in the coding sequences in order to adapt to the new code. Codon *i *will now be used at sites of type *α *if  = *B*^*new *^(*α*). Hence, after adaptation to the new code, the codon frequencies and code cost are:

(13)

(14)

where the new values are used both in the cost function and the codon frequencies. The change in the cost between the new and old codes is ΔΦ = Φ^new^-Φ^cur^. The more negative ΔΦ, the greater the increase in fitness due to the addition of the new amino acid. ΔΦ will be negative for adding almost any amino acid in almost any position of the code in the early stages of code evolution because of the decrease in the Φ_0 _term, as discussed in the previous section. However, this decrease in Φ only applies after many mutations have occurred in the coding sequences and the sequences have adapted to the new code. The difference in cost immediately after the code has changed and before the genes have adapted to the new code is δΦ = Φ^int^-Φ^cur^. The value of δΦ is strongly dependent on which new amino acid is added in which position. If an amino acid is added in a random position, δΦ is likely to be positive, because the codons previously specified the best available amino acid for the site, whereas they now specify a new randomly added amino acid. In other words, there is a barrier to adding an amino acid in a random position because it disrupts the protein sequences that were being synthesized using the old code. If δΦ is positive, selection will tend to prevent the addition of the new amino acid to the code because the organism that first tries out the new code will initially have a lower fitness than the rest of the population. Note that δΦ is likely to be positive even if ΔΦ is negative, so the short-term cost will prevent addition of the new amino acid even if there would be a long-term benefit after the genes adapted to the new code.

Now we reach one of the most important points in this paper. If the new amino acid is added in a position that causes minimal disruption to genes coded by the existing code, it is possible for both δΦ and ΔΦ to be negative. In this case, natural selection will favour the addition of the new amino acid because the organism that tries out the new code will have an increased fitness initially, and an even further increased fitness after its genes adapt to the new code. To see this, let *a *be the new amino acid. Before the addition of *a*, the most similar amino acid in the current code was *b *= *B*^*cur*^(*a*). At sites of type *a*, codons coding for *b *were used because these were the best available. Now suppose some of the codons of *b *are reassigned to *a*. Codons that code for *a *are now used at sites of type *a*. These codons are now in the right place without any mutation in the gene sequence; hence cost is reduced at sites of type *a*. It should be remembered, however, that there are also sites of type *b *at which codons for *b *were already being used in the right place before the code was changed. Thus, when some of these codons are given to *a*, the cost at sites of type *b *increases initially. Furthermore, the addition of *a *can cause changes in costs at sites that are neither type *a *or type *b*. So the value of δΦ depends on a mixture of all these effects. The main goal of the rest of this paper is to demonstrate that there are indeed some cases for which δΦ is negative, and that the evolution of the code is likely to follow the pathways for which δΦ is negative.

Several aspects of the above theory are similar to that of Ardell and Sella [26]. These authors also consider a set of sites of different types and calculate frequencies of each codon and each site type. These authors calculate the invasion fitness of a variant code, which is the fitness of an individual using the variant code to translate a message that has the expected equilibrium codon usage of the established code. The variant code can spread if the invasion fitness is higher than the fitness of the established code. This is equivalent to our criterion that δΦ is negative. However, the changes in the code used in [26] correspond to reduction of ambiguity of codon blocks that previously coded for more than one amino acid as well as reassignment of a codon block from one single amino acid to another. Also, code fitness depends on mutational load in Ardell and Sella's model; whereas in our case it only depends on translational error. We have ignored mutation because of the expectation that if the code evolved in a late-stage RNA world, the organism must already have evolved an efficient means of accurate replication of long RNAs, so the mutation rate would already be small. On the other hand, if the translation process is new, it is likely to be inaccurate initially. Practically, it is much more complex to deal with mutations than translational error. The method of [26] requires calculating the quasispecies distribution that describes mutation-selection balance. This is an infinite-population approximation. A full treatment of mutation would need to introduce finite population size and fixation rates, as in population genetics theory. We showed above that leading term Φ_0 _in the code cost does not depend on *ε*. If mutation rate, *u*, were also added to the theory, then in the limit of small *u*, Φ_0 _would not depend on *u *either. As it is the changes in Φ_0 _that are the major component of δΦ and ΔΦ, we expect that ignoring mutational load will affect the conclusions very little.

### The earliest amino acids and the four-column code

Before we can consider pathways of code evolution, we need to consider the starting point. It has been noted that there is a strong correspondence between the amino acids found in meteorites and Miller-Urey experiments [41,42] and it has been suggested that the amino acids that are found in these situations are the ones most likely to have been early additions to the genetic code [19,20]. Trifonov [43] assembled many papers in which a huge variety of criteria had been used to predict the order of addition of amino acids to the code, and used these to obtain a consensus rank order. This ranking appears useful to us, but we were concerned that it incorporates many very speculative criteria. Therefore, we repeated a similar procedure using only criteria based on measured concentrations of amino acids in non-biological contexts [23,24]. The order that emerges is very similar to that of Trifonov [43]. The criteria we use include prebiotic synthesis experiments of the Miller-Urey type, observed frequencies in carbonaceous chondrite meteorites, synthesis in hydrothermal vent conditions, experiments mimicking the chemistry on icy dust grains in protoplanetary disks, and a variety of other attempts at prebiotic chemical synthesis. These different experiments are related to completely different theories about the mechanisms and locations of amino acid formation – on Earth versus outside Earth, high versus low temperature, *etc*. Nevertheless, there is a surprising consensus on *which *amino acids are easy to form, even if there is no agreement at all on where and how. The following 10 amino acids are found in many of these experiments, and these can be ranked in order of decreasing relative concentrations, as follows:

Gly, Ala, Asp, Glu, Val, Ser, Ile, Leu, Pro, Thr.

We refer to these as the 'early' amino acids. We presume that these were available in the environment at the time the first code arose, and that they could be incorporated into the code at an early stage. The remaining 10 biological amino acids are not seen in these experiments related to prebiotic conditions. We refer to these as 'late'. We presume that these are not easy to form non-biologically, and that they require biochemical pathways to synthesize them inside organisms. Thus, they could only be added to the code at a later stage, after biochemical synthesis pathways evolved. We agree with proponents of the coevolution theory of the code [19-22] that the evolution of biochemical synthesis pathways alongside the development of the code is important. The top 10 according to our procedure are exactly those in the Miller experiments, however, we wish to remain agnostic on the mechanism of amino acid formation because completely different mechanisms give rise to essentially the same amino acids. The reason for this appears to be thermodynamics. Higgs and Pudritz [23,24] showed that the rank order for the 10 early amino acids correlates extremely well with the free energy of formation of the amino acids. The amino acids that are thermodynamically least costly to form are at the top of the list.

**Figure 2:**
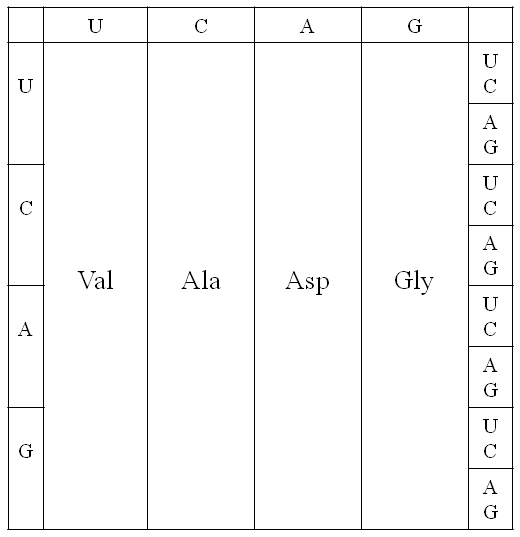
**Proposed four-column structure of the earliest genetic code**.

It is remarkable that the top 5 amino acids on our list (i*.*e. those with highest prebiotic concentrations) are precisely those that occupy codons with G at first position. This leads us to propose a very early version of the code that used only these GNN codons. This same early stage of the code is also proposed in a recent version of the coevolution theory [22], and this also forms the starting point for several much earlier treatments of genetic code evolution [17,44-47]. It has also been proposed that that a regular pattern of G's at first position could have been important in keeping the early ribosome in frame [48]. However, if three quarters of the codons were unassigned, then all mutations occurring at 1^st ^position would render the gene non-functional or impossible to translate. We therefore propose that the code rapidly expanded to give the four-column structure in Figure 2. As both Asp and Glu are in our top 5 early amino acids, it is possible that the third column could have been Glu instead of Asp, or that there was a mixture of Asp and Glu codons in this column, or that this column coded both Asp and Glu ambiguously. For concreteness in Figure 2, we have taken the simplest possibility, which is that this column coded only for Asp.

The four-column code is a triplet code, although only the middle base in the codon specifies any information. Although it is possible to code for only four amino acids with codons of length two (or even one) base, it is unlikely that this was ever the case, because the evolution of such a code to a triplet code would require the complete reinvention of the mechanism by which the ribosome shifts along the mRNA and the complete rewriting of all the gene sequences that were written in the two-base or one-base code. Furthermore, it is likely that the earliest tRNAs – or at least the anticodon loop of these tRNAs – had the same structure as modern tRNAs. The structure of the anticodon loop is thermodynamically stable and has remained evolutionarily stable in modern tRNAs of all types. The codon-anticodon interaction involves the bases at all three positions. It is unlikely that stable codon-anticodon recognition and discrimination could have been achieved with fewer than three bases.

The typical set of tRNA genes found in modern prokaryotes is such that there is at least one tRNA gene for every pair of codons: a tRNA with a G (or modified G) at the wobble position pairs with codons ending U and C, and a tRNA with U (or modified U) at the wobble position pairs with codons ending A and G [49,50]. In mitochondria, there are many cases where a single tRNA with wobble-U pairs with all four codons in a four-codon block, however, it seems clear that these are derived from bacterial ancestors that used two different tRNAs for every four-codon block. There are also a few cases where bacteria use only one tRNA for a four-codon block, but these are probably also due to tRNA gene deletions, as they occur principally in parasitic or endosymbiotic bacteria with very reduced genome sizes. Thus, under the assumption that each tRNA paired with two codons, it would have required 32 different tRNAs to translate the four-column code – 8 for each amino acid.

If the GNN code existed before the four-column code, as suggested above, then all tRNAs would have had C at the 3^rd ^anticodon position (which matches the 1^st ^codon position). It is straightforward for the GNN code to progress to the four column code by duplication of these tRNAs and mutation of the C to any of the other bases. The 8 tRNAs for each amino acid would then have differed from each other at the 1^st ^and 3^rd ^anticodon positions, but may have been almost identical in the rest of the sequence. The tRNAs must have been 'rechargeable', as they are now. After each amino acyl-tRNA has had its amino acid used in protein synthesis, it must be recognized by an amino acyl-tRNA synthetase and recharged with the correct amino acid. The synthetases are proteins in modern organisms, but as the earliest stages of code evolution occurred in the RNA world, we must suppose that there were synthetase ribozymes that did the charging. Correct charging requires accurate molecular recognition that distinguishes one tRNA from another. The proposed four-column code makes this relatively easy. If early synthetase ribozymes used the anticodon to recognize the correct tRNA (as do many modern synthetase enzymes), then all they would need to do is recognize the middle base of the anticodon. All tRNAs with the same middle base would be charged with the same amino acid. Thus, possibly only four synthetase ribozymes were required at this stage.

The proposed four-column code has one further important advantage – it minimizes the effects of translational error. If the relative rates of different types of error are as in Equation (5), then the total error rate of any codon in the four-column code is 0.7*ε*, because only 2^nd ^position errors have an effect. This may be compared with a codon in a two-codon family in the modern code, in which the total error rate is 4.7*ε*. The value of *ε *is a property of the ribosome. Early ribosomes were probably error-prone. It would therefore be important to begin with a code with a simple structure in which the effects of errors were minimized. There would also have been selection on rRNA to reduce the error rate of the ribosome at the same time as the code was evolving, and this may have helped to minimize the increasing number of errors that would be inevitable as the number of amino acids in the code increased and the codon blocks got smaller.

Although the relative rates of errors of different types may not be precisely as in Equation (5), the reason that the error rate at 2^nd ^position is lower than the other two positions is easy to see from the point of view of RNA structure. It is known that a large proportion of the free energy associated with helix formation is due to stacking of neighbouring base pairs in a helix rather that hydrogen bonding of single pairs between the two strands. For this reason, single base pairs are unstable in the standard set of energy rules used for RNA secondary structure prediction (see [51] and references therein). If a mismatch occurred at the 2^nd ^position in the codon-anticodon interaction, this would leave two unstacked single pairs; therefore this would be very unstable and the corresponding error would never occur. If the mismatch occurred at 1^st ^or 3^rd ^position, this would still leave two stacked pairs. This might be marginally stable for sufficiently long for the ribosome to make an appreciable error rate. Given the importance of the middle base in the codon-anticodon interaction, it seems logical that it should be the middle base that contains the coding information in the four column code that we propose.

In summary, the thermodynamics of amino acid synthesis, the simplicity of tRNA charging, and the low rate of translational error all point to the four-column code as a good candidate for an early stage in genetic code evolution. We therefore treat this code as a starting point in what follows.

### Evolution of the four-column code by subdivision of codon blocks

**Figure 3:**
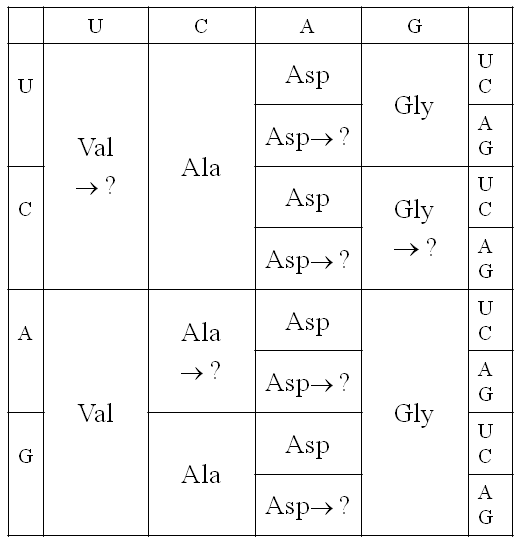
**Possible evolution of the four-column code via subdivision of codon blocks**. One example of a possible subdivision process is illustrated in each column of the code.

Figure 3 shows several possible steps by which the four-column code could have evolved via subdivision. One possible example of subdivision has been chosen in each column. In column 1, we consider the reassignment of the 8 codons UUN and CUN from Val to some other amino acid, and we aim to determine which other amino acids might be added to this position. It would appear that, in fact, Leu was added to this position, and we wish to see whether the theory can predict this. In order to reassign these codons, there must have been some change to the amino acyl-tRNA synthetase ribozyme that was charging these tRNAs. The tRNAs for the UUN and CUN codons would have had A and G at the third anticodon position, whereas those for AUN and GUN would have had U and C. Hence, the new synthetase needs to evolve the ability to distinguish between purines and pyrimidines at the 3^rd ^anticodon position (in addition to discrimination of the 2^nd ^position base, which it does already). We suppose that the original synthetase for the Val column duplicates and that the two synthetases diverge evolutionarily and specialize to different amino acids and different codons. In this way the organism can 'try out' using a new amino acid in the UUN and CUN codon blocks.

In column 2, the ACN block was chosen as an example. This is now occupied by Thr. To distinguish this four-codon block would require the evolution of a synthetase that recognized U at the 3^rd ^anticodon position. The example chosen in column 4 is CGN, which is now Arg. This could have occurred by a similar kind of evolution of the synthetases. In Column 3, we considered a possible subdivision into 8 blocks of 2 codons. This would only require a new synthetase that discriminates between purines and pyrimidines at the 1^st ^anticodon position (i*.*e. the wobble position). Creation of 8 blocks of 2 in column 3 is thus no more difficult than creation of 2 blocks of 8 in column 1. The synthetase simply learns to discriminate a different base in the anticodon. We will consider the possible addition of Glu to the 4 blocks of 2 that are reassigned (as shown in Figure 3). We know that Glu occupies GAR in the final code, but CAR and AAR are occupied by Gln and Lys, both of which are similar to Glu (see Figure 1). This points ahead to what might have happened at a later stage of code evolution. As a first step, however, we will simply look at addition of Glu.

We acknowledge immediately that we have no reason to say why a particular pattern of subdivision into 8's, 4's or 2's should have occurred in one column rather than another. At this point, we have simply picked possibilities that look like they are consistent with the positions of the codon blocks in the final code. The proposed method for trying out a new amino acid in the code depends on the evolution of a new synthetase ribozyme that handles an amino acid that was not previously in the code. It does not necessarily require evolution of the tRNAs. This is in contrast to what occurs in codon reassignments in modern codes, where the reassignment is constrained to keep the same 20 amino acids. In the modern reassignments, it is usually the tRNA that changes by an anticodon mutation or a base modification so that it gains the ability to pair with a new codon [10,11]. The synthetase enzyme does not change, because it is important that the mutated or modified tRNA is still charged with its original amino acid.

**Figure 4:**
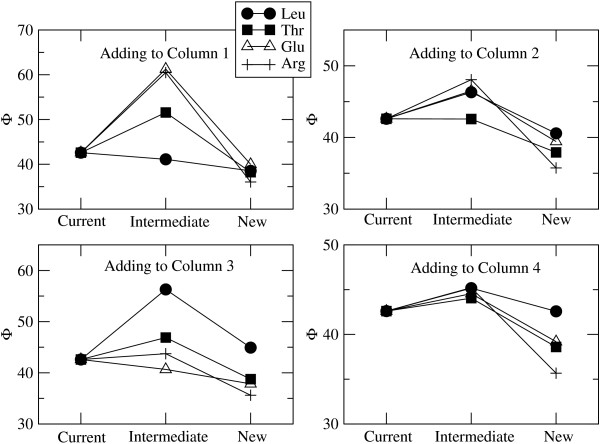
**Changes in code cost function Φ when attempting to add the amino acids Leu, Thr, Glu and Arg to the four different positions illustrated in Figure 3**. Current, Intermediate and New denote the value of Φ before reassignment, immediately after reassignment, and considerably after reassignment when the genes have adapted to the new code. The difference *δ *Φ = Φ_int_-Φ_cur _is the selective barrier against addition of the new amino acid. The figure shows that it is favourable to add Leu in column 1, Thr in column 2, and Glu in column 3, but it is not favourable to add Arg in any of the columns.

The four amino acids that appear to have been added in the positions shown in Figure 3 are Leu, Thr, Glu and Arg. We will therefore consider what happens to the cost of the code when we try adding each of these amino acids in each of the four positions. Figure 4 shows the cost of the current code (Φ_cur _in equation 11), the intermediate state when the new amino acid is first tried out (Φ_int _in equation 12), and the new code after the codons have adapted to the code (Φ_new _in equation 14). The current code is the four-column code in Figure 2 in each case; therefore Φ_cur _is the same in each case. These examples were done with *ε *= 0.05, which we consider to be a relatively large error rate.

In column 1, it can be seen that ΔΦ is negative for addition of all four of these amino acids, but δΦ is negative only for Leu. Thus it would pay to add any of them in the long term, but the change that causes the reassignment is only favoured selectively in the short term for Leu. In column 2, δΦ is slightly negative for Thr, but substantially positive for the others. In column 3, δΦ is negative for Glu and positive for the others. Thus in the first three columns, selection favours the addition of the correct amino acid in each case. In column 4, however, δΦ is positive for all four amino acids. Selection does not favour the addition of Arg in column 4 or in any of the other positions considered in Figure 3. Notice also that ΔΦ for Arg is the most negative of the four amino acids considered for each of the four columns. There is a lot to gain from adding Arg because it is very different from any of the amino acids already present in the code, and by adding it, the diversity of possible protein sequences would be increased more than by addition of Leu, Thr or Glu. However, because Arg is very different, it is disruptive to the existing sequences and therefore there is a large barrier preventing its addition to the code.

Figure 4 illustrates the fact that there is a selective barrier for addition of most amino acids in most positions, but that the barrier can be negative for particular amino acids in particular positions. We now consider the details of this more carefully. Table 2 shows the values of δΦ and ΔΦ for the attempted addition of all 20 amino acids to the 8-codon block in column 1. Results are shown when error is present (*ε *= 0.05) and when error is absent (*ε *= 0). The amino acids are listed in order of increasing δΦ when error is present. Leu has the most negative δΦ, i*.*e. Leu is the amino acid whose addition at this position is most strongly favoured by selection. Of course, Leu is exactly the amino acid that is added at this position in the real code. The addition of Ile is also favourable. For Val, both δΦ and ΔΦ are zero, because these codons are already assigned to Val. The other amino acids all have positive δΦ. As the other three columns are occupied by a single amino acid, it makes no difference which position in column 1 is reassigned. If the calculation is repeated with a block of 4 or 2 codons anywhere in column 1, then the result is that δΦ is negative for Leu and Ile only. In particular, if the AUN block (which is Ile in the standard code) is considered, δΦ is negative for Ile. Thus selection favours the addition of both Leu and Ile to their correct positions.

**Table 2:** Barriers (δΦ) and net changes in cost (ΔΦ) for addition of amino acids to an 8-codon block in column 1.

	*ε *= 0.05		*ε *= 0	
	δΦ	ΔΦ	δΦ	ΔΦ

Leu	-1.49	-4.07	-1.56	-4.54
Ile	-0.99	-3.26	-1.07	-3.69
Val	0.00	0.00	0.00	0.00
Phe	1.09	-4.02	1.07	-4.84
Met	2.00	-3.83	2.11	-4.63
Cys	6.60	0.01	6.92	-0.67
Tyr	6.68	-4.66	6.94	-6.17
Trp	8.33	-1.72	8.50	-3.21
Thr	8.97	-4.38	9.55	-5.98
Pro	11.20	-3.69	11.84	-5.70
His	11.51	-4.67	12.05	-6.79
Ala	11.67	1.19	12.33	0.00
Gln	14.49	-6.81	15.16	-9.69
Ser	15.25	-1.36	16.05	-3.36
Asn	16.73	-3.65	17.51	-6.41
Arg	17.90	-6.56	18.52	-9.69
Glu	18.68	-2.61	19.44	-5.71
Lys	19.25	-6.51	19.96	-9.71
Asp	20.70	2.50	21.54	0.00
Gly	21.60	2.30	22.48	0.00

Table 2 also shows that the two amino acids with the smallest positive barrier are Phe and Met, which also are found in column 1 in the standard code. These two amino acids are probably later additions; therefore we will return to them later.

It can be seen that there is little difference between the results with *ε *= 0.05 and *ε *= 0. This is because there is a big potential gain in the leading cost term (Φ_0 _in equation 9), which is not affected by translational error. One difference that is apparent is that when *ε *= 0, ΔΦ = 0 for Ala, Asp and Gly, which are already in the code elsewhere, whereas ΔΦ is positive for these amino acids when *ε *is non-zero. This is because there is no gain in the Φ_0 _term for adding an amino acid to column 1 that is already present elsewhere, but the translational error term is made worse by doing this. The fact that the error term makes little difference is good news, because this term depends on quantities that we do not know precisely, such as the relative rates of errors of different types. Thus, the conclusion that the addition of Leu and Ile into column 1 is selectively favourable is valid, despite the uncertainty of these details.

Table 3 considers possible additions to the other three columns in the positions illustrated in Figure 3. For simplicity, only δΦ is shown in these tables, because ΔΦ tells us little about the evolutionary dynamics. For additions to the ACN box, which is Thr in the standard code, the only amino acid with negative δΦ is in fact Thr. The two smallest positive barriers are for Ser and Pro, which are also found in column 2. Thr, Pro and Ser are, of course, the amino acids that need to be added into column 2. The addition of these three amino acids is thus either selectively favourable or can be achieved by overcoming only a small positive barrier. We will consider small positive barriers again below.

**Table 3:** Barriers (δΦ) for addition of amino acids to codon blocks in columns 2, 3 and 4 in the positions indicated in Figure 3.

Col 2 (4 codons)			Col 3 (4 × 2 codons)			Col 4 (4 codons)		
	*ε * = 0.05	*ε *= 0		*ε * = 0.05	*ε *= 0		*ε * = 0.05	*ε *= 0

Thr	-0.03	0.01	Glu	-1.94	-2.02	Gly	0.00	0.00
Ala	0.00	0.00	Gln	-1.89	-1.93	Ser	0.77	0.92
Ser	0.10	0.06	Asn	-1.65	-1.67	Ala	0.98	1.09
Pro	0.53	0.57	Lys	-0.66	-0.73	Asn	1.44	1.69
Asn	2.10	2.13	Asp	0.00	0.00	Thr	1.45	1.65
Cys	2.27	2.41	Arg	1.13	1.11	Pro	1.62	1.83
Gln	2.63	2.72	His	1.22	1.31	Asp	1.65	1.87
Gly	2.65	2.61	Pro	3.81	4.03	Cys	1.75	1.84
His	2.75	2.87	Thr	4.28	4.54	Gln	1.85	2.13
Met	3.23	3.47	Ser	5.42	5.73	His	1.86	2.10
Val	3.48	3.74	Tyr	6.28	6.53	Glu	1.95	2.21
Asp	3.62	3.65	Ala	9.33	9.80	Met	2.29	2.45
Leu	3.71	4.00	Met	9.89	10.32	Lys	2.42	2.68
Glu	3.89	3.96	Trp	11.65	12.04	Val	2.46	2.58
Tyr	3.97	4.18	Gly	12.10	12.64	Tyr	2.54	2.75
Ile	4.21	4.51	Phe	12.94	13.45	Leu	2.57	2.70
Phe	4.59	4.88	Cys	13.43	14.00	Arg	2.61	2.85
Lys	4.79	4.89	Leu	13.68	14.25	Ile	2.69	2.80
Arg	5.46	5.59	Val	14.26	14.85	Phe	2.78	2.91
Trp	6.20	6.46	Ile	15.15	15.76	Trp	3.25	3.39

For the calculations shown in Table 3, it was supposed that the other columns were still uniform, i*.*e. the whole of column 1 was still Val. In this case it makes no difference whether we consider addition to the UCN or CCN block instead of the ACN block. The assignments of Thr, Pro and Ser in column 2 may have occurred before the assignments of Leu and Ile in column 1. According to current biosynthetic pathways [20,22], Thr is a precursor of Ile, so it might make more sense to add Thr first. However, Ser, Ile, Leu, Pro and Thr, which are assigned to columns 1 and 2, are all found non-biologically to some extent. Therefore they appear in our list of 10 early amino acids. If these amino acids were present in sufficient quantities in the environment, they could have been incorporated into the code before biosynthetic pathways evolved. Note that, if *ε *= 0, then the columns are completely independent, so the order of addition of these amino acids to columns 1 and 2 makes no difference to the calculation. If *ε *is non-zero, it only makes a small difference, because the 2^nd^-position error rate is small. Furthermore, Val, Leu and Ile are all similar, so when we consider addition of Thr to the ACN block, for example, it only makes a very small difference whether the AUN block is assigned to Val, Leu or Ile at this point.

For additions to the four 2-codon blocks in column 3, Table 3 shows that the most negative δΦ is for Glu, exactly as expected. δΦ is also negative for Gln, Asn and Lys. These amino acids are also found in column 3 in the standard code, so this makes sense. However, these three are late amino acids, so we will presume that Glu was added at this early stage, and we will return to the other three amino acids at a later stage. The splitting of column four into 8 blocks of 2 codons in this way is speculative, but is appealing because, in one step, it creates the structure of 8 blocks that is a feature of column 3 in the standard code. We have explained above, why creation of 8 blocks of 2 is no more difficult than creation of 2 blocks of 8. An alternative would be that only the two GAR codons were reassigned at this stage. The same amino acids have negative δΦ if only GAR codons are considered. Thus, either way, this theory successfully predicts that Glu can be added into its correct position.

Table 3 also considers additions to the CGN block in column 4. No amino acid has negative δΦ. The smallest positive barrier is for Ser, which is suggestive because Ser does occur in column 4 in the standard code. The barrier is pretty large for all the other amino acids, including Arg, which is found in the CGN position in the standard code. Thus, we conclude is that it is not selectively favourable to add any new amino acids to column 4 at this stage. It should also be noted that Arg, Cys, and Trp (which need to be added to column 4 at some stage) are all late amino acids, so they may not have been around at this early stage of code evolution in any case.

### Later stages of code evolution

**Figure 5:**
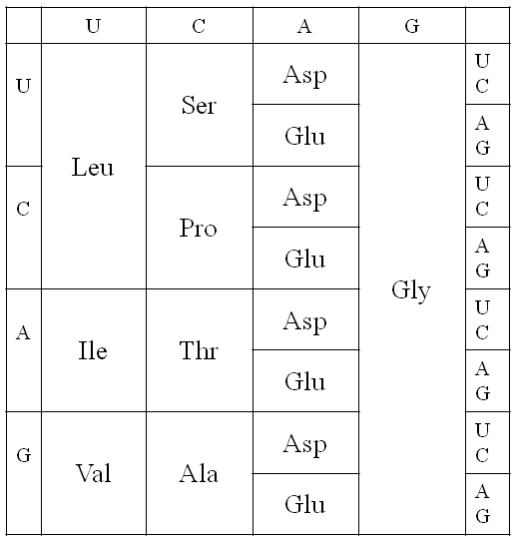
**Proposed structure of the code after the 10 early amino acids have been added**.

In the previous section, it was demonstrated that the amino acids Ser, Ile, Leu, Pro and Thr, can all be added to their correct positions in the code either by positive selection (negative δΦ) or by overcoming only a small selective barrier. It therefore seems reasonable to propose a 10 amino acid code shown in Figure 5, which is an intermediate stage of code evolution that incorporates all 10 of the amino acids that we classed as early [23,24]. We will now consider possible evolution of the code from this point on.

Table 4 shows values of δΦ and ΔΦ for attempted reassignment of the two UUY codons from Leu to other amino acids. There are no amino acids with negative δΦ. The smallest positive barriers are for Ile and Phe. The standard code has Phe in this position. For the amino acids that are already in the code, including Ile, ΔΦ is positive (not shown in the table). This is because assigning UUY to these amino acids gains nothing in improved protein diversity but increases translational error with respect to the 10 amino acid code. Thus, there is no driving force for adding Ile in the UUY position. On the other hand ΔΦ is negative for adding Phe. So the theory predicts that Phe is the easiest amino acid to add at this point that is not already in the code, but suggests that there is a small barrier to overcome during the reassignment. The next easiest after Phe is Met, and Met is also found in column 1. We repeated the analysis with the AUG codon, which is Met in the standard code, and found similar results, i*.*e. the two amino acids with the smallest positive barriers that are not already included in the code are Phe and Met.

**Table 4:** Barriers (δΦ) for addition of amino acids to four different 2-codon blocks beginning from the 10 amino acid code in Figure 5.

UUY (Phe)		AAY (Asn)		AAR (Lys)		AGR (Arg)	
Leu	0.00	Asn	-0.02	Lys	-0.27	Gly	0.00
Ile	0.32	Asp	0.00	Gln	-0.16	Ser	0.36
Phe	0.41	Glu	0.23	Glu	0.00	Ala	0.48
Met	0.73	Gln	0.53	Arg	0.24	Asn	0.68
Val	0.75	Lys	1.09	Asn	0.46	Thr	0.69
Tyr	2.17	His	1.11	His	1.04	Pro	0.77
Cys	2.21	Ser	1.20	Asp	1.13	Asp	0.80
Trp	2.37	Pro	1.21	Pro	1.89	Gln	0.86
Thr	2.83	Thr	1.27	Thr	2.10	Cys	0.86
His	3.38	Arg	1.55	Tyr	2.27	His	0.88
Pro	3.41	Ala	1.92	Ser	2.66	Glu	0.92
Ala	3.55	Tyr	2.11	Met	3.62	Met	1.11
Gln	4.18	Gly	2.20	Ala	3.84	Lys	1.14
Ser	4.39	Met	2.50	Trp	3.86	Val	1.22
Asn	4.76	Cys	2.79	Phe	4.48	Tyr	1.22
Arg	4.98	Leu	3.05	Leu	4.81	Arg	1.23
Glu	5.24	Phe	3.06	Gly	4.81	Leu	1.26
Lys	5.37	Val	3.06	Cys	4.99	Ile	1.32
Asp	5.77	Trp	3.06	Val	5.06	Phe	1.36
Gly	6.02	Ile	3.29	Ile	5.26	Trp	1.58

The amino acid assigned to each of the codon blocks in the standard genetic code is indicated at the top of the table. *ε *= 0.05 in all cases.

Table 4 shows three further examples of reassignment of two codon blocks from the 10 amino acid code. For the AAY block, which is Asn in the final code, the only amino acid with negative δΦ is Asn. For the AAR block, which is Lys in the final code, the most negative δΦ occurs for Lys, and Gln also has negative δΦ. The calculation for the CAR block, which is currently Gln, is not shown because it is almost identical to the AAR block, i*.*e. both Lys and Gln have negative δΦ for addition to the CAR block. Thus, the theory predicts that the addition of Asn, Lys and Gln into their current positions in column 3 are all selectively favourable. Finally, Table 4 considers addition the AGR block, which is currently Arg. These results are similar to those in Table 3 for the CGN block. There are no additions that are favourable. The general conclusion of the last two sections is that the theory is able to predict the evolution of the first three columns of code very well, but does not apply well to column 4.

### Discussion of puzzles remaining

When δΦ is negative, selection should immediately favour codon reassignment by the subdivision process. The results above show many cases where amino acids can be directly added into their current positions with negative δΦ (Leu, Ile, Thr, Glu, Asn, Lys, Gln). However, there are also several examples where the amino acid in the current code is the easiest one to add at that position but there is a slightly positive δΦ to overcome (Ser, Pro, Phe, Met). The code cost is dependent to some extent on the choice of amino acid distance function, the frequencies of sites of different types, and the relative rates of errors of different types. It is therefore possible that there is some other slightly different choice of these parameters that would turn these slightly positive δΦ's into slightly negative ones. However, it would be extremely fortuitous if there were some set of parameters for which δΦ turned out to be negative in every case. It therefore seems important to consider how small positive barriers might be overcome.

The theory given above is deterministic. It assumes that all types of sites occur in the genome and that all types of errors occur with given probabilities. It thus ignores fluctuations that would occur in finite size genomes. We suggest that small positive barriers can easily be turned into small negative ones due to fluctuations in finite size genomes. Consider the reassignment of UUY from Leu to Phe (Table 4). The theory assumes that there are some sites where the optimal amino acid is Leu and some where it is Phe. The most similar amino acid to Phe in the 10 amino acid code is Leu; therefore Leu codons are used at Phe-type sites as well as Leu-type sites. The deterministic theory assumes that all 8 codons in the UUN and CUN blocks are used with equal frequency at both types of sites. If the UUY codons are reassigned to Phe, this is advantageous where these codons occur at Phe-type sites and deleterious where they occur at Leu-type sites. In a small genome however, there will only be a small number of sites of each type. It is therefore possible that by chance, the number of UUY codons at Phe-type sites will be slightly higher than the number at Leu-type sites. This will tip the balance, and mean that the variant code will be selectively favoured at the point when it first arises. This argument only works because Leu and Phe are relatively similar and because Leu codons were used at Phe-type sites. If one tries to assign a completely different amino acid to UUY – say Lys – this will not occur even in a small genome because the UUY Leu codons would not be used in Lys-type sites prior to the origin of the variant code that incorporates Lys. Thus, a variant code that assigned UUY to Lys would be selected against, even in a small genome.

One piece of evidence that led us to propose the four-column code is that the five earliest amino acids (Gly, Ala, Asp, Glu and Val) are assigned to bottom-row codons (GNN), and this naturally suggests a code in which the 2^nd ^position is the one which specifies the coding information, as discussed above. However, if all the codons in a given column are equivalent in the four-column code (Figure 2), there is no logical reason why the earliest amino acids should remain on the bottom row. For example, it would be equally likely for the AUN and GUN codons to be reassigned to Leu, the CUN to Ile, and for Val to retain the UUN codons. The fact that the earliest amino acids remain on the bottom row means that there must be some special significance of the GNN codons that is retained throughout the early period of code evolution from the four-column code (Figure 2) to the 10 amino acid code (Figure 5). We suggest that this is to do with codon bias. It will always be easier to reassign codons that are rarer, especially if the barrier is slightly positive (as in the previous paragraph). Thus if GNN codons are preferred with respect to the other synonymous codons in the same column, this provides a reason why the earliest amino acids retain the frequent GNN codons and the other rarer codons are reassigned. If 1^st ^position G was important in keeping the early ribosome in frame, as suggested above, then it may have been advantageous to retain a G at first position wherever possible. Alternatively, if the first tRNAs were those that translated the GNN codons, it is possible that these tRNAs remained at higher concentrations in the cell even after tRNAs for the other codons arose. In that case translational selection would favour codons that matched the most frequent tRNAs and would therefore maintain a bias in favour of GNN codons. Whatever the reason, it is necessary for there to be some asymmetry of the code in favour of GNN codons, or else the earliest amino acids would have ended up randomly positioned in their respective columns, and the compelling pattern of early amino acids in GNN codons would not be retained in the final code.

The obvious problem for this theory is that it does not seem to apply well to column 4. Addition of Arg, Cys and Trp all require overcoming relatively large barriers in the calculations above. This may be partially understandable, however. Although, Gly is the most frequent prebiotic amino acid, and therefore is assumed to be included in the earliest code, it is an outlier in physical property space, as shown in Figure 1. As each new amino acid is added, it is always more similar to at least one of Val, Ala, or Asp than it is it to Gly. Therefore, the new amino acids prefer to join the other three columns and Gly is left all alone in column 4.

By the time we reach the 10 amino acid stage there is a good diversity of amino acids in the code. There is no reason to use Gly as a best alternative to anything else, i*.*e. Gly codons will only be used in Gly-type sites where Gly is really required. This means that codons in the Gly column will be relatively rare, and if there is a bias towards G at first position, then UGN, CGN and AGN codons will be particularly rare. We suggest, therefore that Arg, Cys and Trp were only added to the code at a late stage and that the rareness of these codons and the corresponding fluctuations of codon numbers in finite size genomes would have helped code variants incorporating these amino acids to be selected. An extreme version of the rare-codon idea is that the reassigned codons might have disappeared altogether, in which case there would have been no barrier at all during the reassignment process. This was proposed by Osawa and Jukes [52] as an explanation of codon reassignment in modern variant codes. We have shown that the codon disappearance mechanism is a good explanation of some (but by no means all) the codon reassignments that occur in mitochondrial genomes [11]. By the time Arg, Cys and Trp were added to the code, there was not much alternative but to join column 4 because all the prime positions in the other columns of the code were already taken. Squeezing them into the other columns would have increased the cost of translational errors in these columns. These three amino acids are also outliers in property space, which means that there is a definite benefit to adding them once the gene sequences adapt to the code – i*.*e. ΔΦ is negative. Thus Arg, Cys and Trp are valuable players in the game of protein structure and function, even if they are among the last to be picked for the team.

The results above depend on the choice of amino acid distance function. As polar requirement (PR) has been widely used previously in studies of the genetic code, we also considered a distance function, *d*_*PR*_, that is proportional to the absolute difference in PR. In equation (1), this is equivalent to setting the weight *w*_*k *_= 1 for PR, and *w*_*k *_= 0 for the other properties. We repeated all the above calculations of genetic code evolution using *g = d*_*PR*_. We will comment on a few significant differences.

For the case of addition to column 1 shown in Table 2, δΦ is negative only for Leu and Ile when *g = d*_*W*_, but if *g = d*_*PR*_, δΦ is negative for Leu, Ile, Phe, Met, Cys, Tyr and Trp. These amino acids are all hydrophobic, and they all have similar PR values, although they differ more in some of the other properties. The *d*_*PR *_distance between all these amino acids is small, whereas the *d*_*W *_distance is more effective at discriminating Val, Leu and Ile (which are really very similar) from the remaining hydrophobic amino acids, which are somewhat less similar. Using *d*_*PR *_makes it easier to add Phe and Met to column 1, but it would also predict adding Cys, Tyr and Trp, which do not occur in column 1.

For the column 2 case in Table 3, the chief difference is that δΦ is slightly negative for Pro when *g = d*_*PR*_, whereas it was slightly positive before. δΦ remains slightly negative for Thr and slightly positive for Ser. For column 3, Glu is the only amino acid for which δΦ is negative when *g = d*_*PR*_, whereas it is also negative for Gln, Asn and Lys when *g = d*_*W*_.

For the CGN block in column 4, no amino acid had negative δΦ when *g = d*_*W*_. Somewhat paradoxically, the barrier is negative for Arg when *d*_*PR *_is used. Although it is tempting to claim this as another successful prediction of the theory, it is more likely that it is just an artefact of the PR scale. For some reason Gly and Arg are relatively close on the PR scale, even though they are far apart on the other properties that contribute to *d*_*W *_(see Figure 1). Unless we are prepared to assume that PR is, for some reason, more important than all the other properties, we cannot reasonably claim that Gly and Arg are similar amino acids. Furthermore, we also find that Gln and His have negative barriers for addition into column 4 when *g = d*_*PR*_. Therefore, if we accepted that *d*_*PR *_was a good cost function, then we would predict the addition of Gln and His to column 4, which would be incorrect, because these amino acids appear in column 3 in the standard code.

It seems that, with respect to the amino acids in column 4, the PR scale is 'too good to be true'. In addition to the relative closeness of Gly and Arg according to this scale, Cys and Trp are also very close to each other and not that far from Gly. In contrast, Figure 1 makes it clear that all these amino acids are completely different from one another according to *d*_*W*_. One reason why the *f *value for randomly reshuffled codes is lower when PR is used than with other single properties is because of the unrealistic closeness of the amino acids in column 4 according to the PR scale. In general, we consider the weighted distance function *d*_*W *_to be a much better cost function than *d*_*PR *_and therefore the conclusions we draw in this paper are based on the use of *d*_*W*_.

We now turn to another mysterious aspect of the standard code: stop codons. Stop codons in the standard code seem to be associated with amino acids that are later additions: UGA is a box with Cys and Trp, and UUA and UAG are in a box with Tyr. Tyr is an outlier in physical property space relative to the other amino acids in column 3, and it is probably a late addition. The current method of termination of translation using protein release factors must be a late-evolving feature because the release factors could only have evolved after protein synthesis was possible with a reasonably large repertoire of amino acids. It is possible that the earliest translation mechanism did not require special start and stop codons. The primitive ribosome might simply have bound to one end of the mRNA and continued until it fell off the other end. At this early stage, mRNA molecules might have been separate molecules inherited independently in an organism of the RNA world, rather than transcripts from a large genome sequence. Thus, there is no reason why mRNAs need have had untranslated regions at either end. Ideas similar to this have also been suggested by Y. Wolf and E. Koonin (personal communication). For the purposes of this paper, we have ignored the possibility of stop codons in the four column code and 10 amino acid codes. If the three current stop codons were treated as stop, it would make little difference to the arguments about which codon reassignments were favourable in the other positions.

In each of the examples considered in Tables 2, 3, 4, we have considered addition of all possible amino acids to the codon block, i*.*e. we have assumed that variant codes could arise that 'try out' any amino acid randomly in the new position, and that the variant code is selected or rejected according to its affect on fitness. At this point we need to consider factors that might cause non-random associations between codons and amino acids that would cause some amino acids to be more likely to be tried out in some positions than others.

The stereochemical theory of code evolution proposes that there were direct interactions between amino acids and nucleotide triplets in the early stages. Recent work using in vitro selection has isolated aptamers that bind to several different amino acids. Statistical tests indicate that amino acid binding sites in the aptamers seem to contain either codon or anticodon triplets for the corresponding amino acid more frequently than by chance [53-55]. Yarus *et al*. [55] consider Ile as an example. If there were a mechanism why Ile molecules should be more likely to be associated with tRNAs for the AUN codon block, this might explain why a variant code with AUN = Ile arose. As we showed above, addition of Ile into any position in column 1 would be favourable, so the stereochemical theory might explain why Ile was added to the AUN block rather than some other block in column 1. We are reluctant to attach too much significance to this however. Stereochemical associations have not been found for all the amino acids, and there is confusion over whether the anticodon or codon triplet should be associated with the binding site. Also it is not clear how evolution would proceed from a direct physical interaction between a triplet and an amino acid to a covalent attachment of an amino acid to the acceptor stem of a tRNA, which is not in contact with the anticodon. Early tRNAs must have required some kind of synthetase ribozyme to charge them. Amino acid binding sites such as those in the aptamers might therefore be found in the synthetases rather than the tRNAs. However, as far as we know, ribozyme synthetases have been replaced by proteins, so this is not testable.

The coevolution theory makes concrete proposals about which amino acids might be tried out at which position in the code [19-22,56]. This is based on the observation that, in many organisms, the synthesis of Asn from Asp occurs while the amino acid is attached to the tRNA. An Asn tRNA is first charged with Asp and then chemically modified into Asn, giving rise to the correctly charged tRNA. The same happens with Gln: the Gln tRNA is first charged with Glu and then Glu is changed to Gln while it is attached to the tRNA. This process fits perfectly with the idea of subdivision of codon blocks. If Asp initially occupies a large codon block, there will be several different tRNAs for Asp. If an enzyme arises that recognizes a subset of these tRNAs and converts Asp to Asn on these tRNAs, then a subset of Asp codons will be reassigned to Asn. In the previous section, we showed that δΦ is negative for the reassignment of Asp codons to Asn and for Glu codons to Gln in column 3 of the code. If the synthesis of Asn and Gln arose initially by this mechanism, this would lead to variant codes that tried out Asn and Gln in positions formerly occupied by Asp and Glu, and these variant codes would be positively selected according to the theory we give above. Coevolution theory therefore agrees with our theory for these two amino acids.

In addition to Asn and Gln, there are several similar examples [22]: Sec can be synthesized from Ser, fMet can be synthesized from Met, and Cys can be synthesized from Ser. These examples are less relevant for early code evolution because Sec and fMet do not have their own codons, and the Cys case seems to be limited to certain organisms and is therefore not likely to be ancestral. Based essentially on the evidence of Asn and Gln, the coevolution theory proposes that all the other amino acid synthesis pathways also arose on tRNAs. Although this is possible, it is a large assumption. Most of the synthesis pathways for the later amino acids involve many steps. These are now carried out by many different protein enzymes and the molecules involved are not associated with tRNAs. The coevolution theory proposes that all these same reaction steps occurred initially on tRNAs and were catalyzed by ribozymes (or by ribozymes with peptide cofactors) in the RNA world. The theory therefore relies on the fact that each of these reaction steps remained the same after the evolution of proteins, that each reaction step became disassociated with tRNAs, and that each ribozyme was replaced by a protein having the same function without changing any of the pathways. The recent extension of the coevolution theory [22] also proposes that the earliest amino acids were synthesized on tRNAs and that these were derived from the central metabolic pathways of glycolysis and the citric acid cycle. If this is true, then these central metabolic pathways must also have been the same in the RNA world. It is possible that this is true but we simply do not know. I have argued this more fully in my comments following the article of Di Giulio [22]. Nevertheless, the logic of the coevolution theory allows strong predictions to be made about the pathways of code evolution. These will be tested in the following section.

### The pathways of genetic code evolution predicted by the coevolution theory are usually not favoured by selection

**Figure 6:**
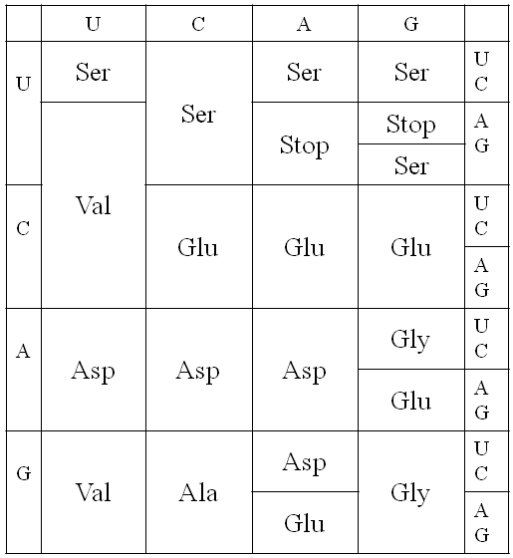
**Early structure of the code predicted by coevolution theory (stage b of Di Giulio and Medugno **[56]).

The coevolution theory proposes that when each new amino acid was added to the code, it took over some of the codons that were previously assigned to its precursor. If one accepts that this is true for all amino acids, and if all the biosynthesis pathways were the same then and they are now, then we can trace each amino acid back to its earliest precursor and deduce the structure of the genetic code at the time when only the earliest precursor amino acids were present. This is done in most detail by Di Giulio and Medugno [56]. The resulting code is shown in Figure 6. This figure corresponds to stage b of Figure 1 of [56]. These authors consider one prior stage in which the Val codons in Figure 6 were assigned to Ala. However, a more recent version shown in Figure 4 of [22], has Val in the position shown. Therefore stage b seems like a good code with which to begin according the coevolution theory.

The code in Figure 6 has the same five earliest amino acids on the bottom row as in our four-column theory, although the pattern of assignments in the rest of the code is different. Stop codons are present in Figure 6. We suggested above that stop codons were a late addition, but the presence or absence of stop codons makes little difference to the arguments in this section. Therefore we will leave the stop codons as they are in [56]. Ser is also included, which is the sixth in our ranking. Thus we are in agreement that this combination of amino acids is perfectly reasonable at an early stage of code evolution. However, in my comments on [22] I have pointed out several reasons why this starting position seems less plausible than the four column code in Figure 1.

Firstly, Di Giulio [22] also argues that only GNN codons were used in the very earliest stage. As discussed previously, the four-column code can arise naturally from the GNN code by duplications of tRNAs and making a single mutations of the 3^rd ^anticodon position to match each of the 1^st ^position bases. On the other hand, the layout in Figure 6 seems pretty random with respect to the GNN code, and there seems no particular reason why the earliest amino acids should have expanded from the bottom row to fill the codon blocks shown.

Secondly, the layout of Figure 6 poses challenges for molecular recognition during the tRNA charging process. We supposed above that there were ribozyme equivalents of amino acyl-tRNA synthetases. Recognition of the set of tRNAs that would be required for the large irregular shaped codon blocks in Figure 6 would either require a sophisticated synthetase that distinguished complex combinations of bases at the three anticodon positions, or would require separate synthetases for the same amino acid that recognized simpler subsets of anticodons. In contrast, the four column code in Figure 4 would only require one synthetase per column that recognized the base at the middle anticodon position. We also argued above that subdivision of the four column code can be achieved by evolving increased specificity of the synthetases. For example, learning to distinguish purines from pyrimidines at the 3^rd ^anticodon position creates two blocks of 8 codons in a column, while learning distinguish purines from pyrimidines at the 1^st ^anticodon position creates 8 blocks of 2 codons. Similar processes of subdivision of codon blocks are not so easy beginning from the code in Figure 6. The mechanism of charging early tRNAs is not clear, and we have no direct evidence that synthetase ribozymes once existed. Nevertheless, if tRNAs were reusable, there must have been some mechanism that distinguished between alternative tRNAs and ensured they were correctly recharged. If amino acid synthesis occurred on the tRNAs (as in the coevolution theory) then synthetases would only be required for the earliest precursor amino acids. However, other ribozymes would be required to catalyze the formation of the product amino acids on the tRNAs, and these ribozymes would need to recognize and distinguish between tRNAs. Therefore, this does not get round the issue of molecular recognition.

Thirdly, the arrangement of amino acids in Figure 6 is poor with respect to translational error because non-synonymous substitutions can arise by errors at all three positions. The four column code is only subject to errors at 2^nd ^position, which are the least frequent. Hence it makes sense that information should be encoded in the 2^nd ^position initially.

Despite the above reservations about the proposed code in Figure 6, let us assume that the early code did indeed have this structure and consider the pathways of subdivision of codon blocks beginning from this point. Table 5 shows values of δΦ obtained using the theory in this paper for the steps of code evolution that were proposed in the coevolution theory. The next step of code evolution from code b to code c of Figure 1 of [56] is that AUN and ACN are reassigned from Asp to Thr, and CCN is reassigned from Glu to Pro. Thr and Pro are relatively early amino acids in our scheme too, therefore we have no problem with the addition of these amino acids at this stage. However, Table 5 shows that selection would not favour the addition of these amino acids at these positions. On the contrary, it would be favourable to add Asn into the AUN and CUN codons formerly occupied by Asp, and favourable to add Lys or Gln to the CCN codons formerly occupied by Glu. The most similar amino acid to Thr and Pro in code b is Ser. It is therefore favourable to reassign some of the Ser codons to these amino acids. As an example, Table 5 shows that it would be favourable to reassign the UUY codons from Ser to either Thr or Pro. Thus, there are several possible pathways with negative δΦ by which code b could evolve, but these are not the pathways predicted by the coevolution theory.

**Table 5:** Barriers (δΦ) for addition of amino acids positions predicted by the coevolution theory. *ε *= 0.05 in all cases.

Current code	b		b		b		c		c		d		d	
Codons changed	AUN+ACN		CCN		UUY		CUN+UUR		AUY+AUA		UUY+UAY		UUR	
Current amino acid	Asp		Glu		Ser		Val		Thr		Ser		Leu	
New amino acid	Asn	-0.29	Gln	-0.48	Thr	-0.57	Leu	-1.41	Thr	0.00	Ser	0.00	Leu	0.00
	Asp	0.00	Lys	-0.23	Pro	-0.30	Ile	-0.98	Tyr	0.44	Thr	0.49	Ile	0.39
	Glu	0.71	Glu	0.00	Ser	0.00	Val	0.00	His	0.59	Ala	0.63	Phe	0.47
	Gln	1.08	Arg	0.21	Ala	0.23	Phe	1.79	Pro	0.62	Pro	0.78	Met	0.83
	Thr	1.65	Asn	0.25	Met	0.47	Met	2.94	Met	0.76	Asn	1.01	Val	0.89
	Ser	1.75	His	0.44	Leu	0.48	Cys	7.18	Gln	1.15	Met	1.21	Tyr	2.51
	Pro	1.79	Asp	1.25	Val	0.54	Tyr	8.96	Leu	1.28	Gln	1.26	Cys	2.58
	His	1.96	Pro	1.43	Cys	0.67	Trp	10.08	Phe	1.34	His	1.27	Trp	2.77
	Ala	2.80	Thr	1.52	His	0.70	Thr	10.13	Val	1.52	Cys	1.29	Thr	3.25
	Lys	3.00	Tyr	1.88	Asn	0.72	Pro	12.48	Asn	1.61	Leu	1.35	His	3.93
	Tyr	3.84	Ser	2.31	Ile	0.74	Ala	12.54	Ile	1.62	Val	1.44	Pro	3.95
	Arg	3.94	Met	3.14	Gln	0.74	His	13.26	Ser	1.66	Gly	1.56	Ala	4.13
	Met	4.00	Ala	3.41	Tyr	0.87	Gln	16.33	Ala	1.66	Ile	1.59	Gln	4.87
	Gly	4.27	Trp	3.77	Phe	0.94	Ser	16.44	Cys	1.70	Tyr	1.67	Ser	5.09
	Cys	4.51	Phe	4.16	Glu	1.56	Asn	18.36	Trp	1.85	Phe	1.69	Asn	5.55
	Leu	4.82	Leu	4.42	Asp	1.63	Arg	20.02	Arg	2.33	Asp	1.71	Arg	5.84
	Val	4.85	Cys	4.62	Gly	1.66	Glu	20.42	Glu	2.36	Glu	1.78	Glu	6.14
	Phe	5.19	Val	4.70	Lys	1.89	Lys	21.19	Lys	2.52	Lys	2.25	Lys	6.30
	Ile	5.36	Gly	4.92	Trp	1.89	Asp	22.31	Asp	2.81	Arg	2.47	Asp	6.77
	Trp	6.03	Ile	4.94	Arg	2.10	Gly	22.78	Gly	3.58	Trp	2.59	Gly	7.05

Let us nevertheless suppose that code c has been created. The next proposed changes that occur between code c and code d of [56] are the reassignment of CUN and UUR from Val to Leu and the reassignment of AUY and AUA from Thr to Ile. Table 5 shows that δΦ is indeed negative for the reassignment from Val to Leu. Essentially the same thing happens in the evolution of the four column code proposed above (Table 2). Table 5 also shows that it would be favourable to reassign some of the Val codons to Ile as well. On the other hand, the coevolution theory predicts that AUY and AUA are reassigned to Ile, and Table 5 shows that δΦ is large and positive for adding Ile in this position.

If we go one step further and assume that code d has been created, the next proposed step from code d to code e of [56] is the reassignment of UUY and AUY from Ser to Phe. Table 5 shows that there is no amino acid that has negative δΦ at this position, but there are many others that have a smaller positive δΦ than Phe. Furthermore, we already saw that it is relatively easy to reassign Leu codons to Phe (Table 4). If we start from code d and try to reassign the UUR codons, then Table 4 shows that Phe is the easiest amino acid to add at this point that is not already in the code, i*.*e. it is much easier to reassign Leu codons to Phe than to reassign Ser codons to Phe, as would be predicted by the coevolution theory.

Thus, we have shown that, with the exception of the reassignment of Val codons to Leu, which occurs in both theories, the early steps of code evolution predicted by the coevolution theory are not favoured by selection. Instead, there are alternative pathways of evolution that *are *favoured by selection that lead away from the layout found in the standard code. Thus, if the code began as in Figure 6, it would not have evolved toward the code we see today, so it seems unlikely that Figure 6 is correct. On the contrary, if the code began with the four column structure proposed in Figure 2, then the steps of evolution that are favoured by selection are precisely those that lead toward today's code, as we have shown above.

We therefore conclude that the central tenet of the coevolution theory, namely that product amino acids always take over the codons of their precursors, is not supported by this analysis of the way natural selection acts during the build-up of the code. This does not mean that biosynthetic pathways of amino acids are irrelevant. It seems clear that the later, more complex, amino acids must have been formed by biochemical reactions inside cells. Therefore, at least for the later amino acids, it must be true that product amino acids were added to the code after their precursors. The order of addition of amino acids that we proposed in the evolution of the four-column code is perfectly consistent with this. However, the position in which a new amino acid is added to the code is not determined by the position of its precursor, but by its physical properties.

## Conclusion

The fact that the standard code minimizes the effects of translational error in comparison to the vast majority of randomly reshuffled codes has been demonstrated convincingly by many different authors using statistical arguments. The observed degree of optimization seems to call for an evolutionary explanation; however there has been very little work that considers the pathways of code evolution in a realistic way. The theory given here uses a formula for code cost that arises straightforwardly from previous cost formulae used for comparing random codes, but develops this into an evolutionary argument by which the pathways of code evolution can be predicted. We have also improved on previous work by introducing a more realistic amino acid distance measure *d*_*W *_that is derived from maximum likelihood fitting of real protein sequence data. The four column code that we propose as an early state is based on evidence about which were the earliest amino acids. It is also supported by the simplicity of the tRNA gene set and charging mechanisms that would be required for this code, and by its robustness to translational error. We have supposed that new amino acids are added to the code when larger codon blocks are subdivided. This theory predicts that new amino acids can be added into positions previously occupied by amino acids with similar properties. This is because, when amino acids are added in this way, there is minimal disruption to the protein sequences that had already evolved under the previous code. This point was recognized already by Crick [14], who stated "The new amino acid should not upset too much the proteins into which it is incorporated. This upset is least likely to happen if the new and the old amino acids are related". The current paper develops this brief statement into a quantitative theory. With this theory, we are able to show that the four column code would naturally evolve under selection toward the standard code used today, and to correctly predict the position of many of the amino acids in the current code. The theory demonstrates that the four-column structure seen in the physical properties of the current code is a remnant of the four-column structure of the earliest code, and it explains why the current code is optimized with respect to translational error, even though translational error is only a secondary factor in the fitness function that determines the direction of code evolution.

## Competing interests

The author declares that they have no competing interests.

## Reviewers' comments

### Reviewer 1

#### David Ardell, School of Natural Sciences, University of California, Merced

In this article, Paul Higgs boldly extends the usual statistical treatments of genetic code optimization into entirely new territory. Rather than a static analysis of code cost and optimization, Higgs explicitly relates cost to fitness, and examines a criterion for genetic code change that is quite close in spirit to our own code invasion criterion in the dynamical theory of code-message coevolution. Unlike in our theory however, Higgs very explicitly addresses the origin of the standard genetic code. He does this by treating codons with three positions, using a well-grounded empirically estimated cost function on extant amino acids, and qualitatively incorporating considerations about early and late amino acid additions to the code, early and late codon assignments, and mechanisms of codon reassignment into his theory. His likelihood estimated partitioning of substitution data in codon mutation and amino acid distance effects is a particularly important contribution to this field.

Also important, Higgs uses his theory to find that he can predict some aspects of the relative placement of specific amino acids to other amino acids in the standard genetic code. And he shows that the specific evolutionary trajectory laid out by Di Giulio and Medugno in the code-metabolism coevolutionary theory would have to overcome significant fitness barriers at each of its steps to have occurred in reality. He therefore shows that it is quite difficult to reconcile their model with the constraint in code evolution to preserve the meaning of protein-coding genes.

The above demonstrates the general utility of Higgs's approach in evaluating models of code change with straight-forward assumptions and a rather simple computational burden. I also think that his arguments for codon reassignment as a major pathway for origin of the standard code are compelling and an improvement on, for example, amino acid swapping between codon blocks.

A major simplification in Higgs's treatment is achieved by removing any explicit modeling of the mutation of codons in protein-coding genes. Selection on codons is also essentialized by assuming that only the "best" encoded amino acids occupy a specific site-type. In his model, selection on codons is very strong and mutation of codons is negligible. Here I think he overlooks that a genome is a population of sites, so that the impact of codon mutation on both the fitness of a genetic code and the relative fitness of code alterations can easily become comparable to that of translational error (Sella and Ardell [27]). Nonetheless, his results are valuable in showing that even without explicitly considering mutation or even translational error, the constraint to translate existing messages can strongly shape codon reassignments in a way that can predict the structure of the standard code.

**Author response**: *Yes, I agree that it would be possible in principle to add mutation to this. In the papers of Sella and Ardell, mutation is added in a deterministic way. It is assumed that there is a mutation-selection balance, so that non-optimal codons of all types appear with small probabilities in all positions. This assumes that the genome is very long and that the population size is infinite, both of which are oversimplifications. It seems to me that a more correct treatment of mutations would be to simulate a finite population of individuals, each with a finite length gene sequence. The sequences of the individuals would become slightly different from one another due to accumulated mutations. The new genetic code variant would arise within one member of the population, and the probability that the variant spreads to fixation would depend on the fitness of this individual relative to the rest of the population. This would be a stochastic simulation that would be very much more complicated than the present treatment, and I have not attempted it. It is relevant, however, because I think this is the explanation of how small δΦ barriers are overcome. In finite-length sequences the numbers of each type of codon occurring at each type of site can fluctuate a lot. For this reason, a new code variant might be slightly favourable when it arises in one individual, even though it would be slightly unfavourable if it arose in an individual where all the codon frequencies were exactly equal to their expected averages. Conceptually, introduction of mutations would smooth out the fitness landscape and add stochastic noise to the direction of evolution that the code takes. The most important aspects of the fitness landscape are determined by the Φ*_0_*term, i.e. the part of the cost function that is non-zero even when both mutation and translational error are zero. For this reason, I do not think that introduction of mutations would make much difference to the major conclusions made above about the likely pathways of evolution of the code*.

It is worth pointing out that amino acid frequencies in modern-day proteins play a perhaps surprisingly large role in Higgs's theory. That is because these frequencies define the frequencies of site-types that codons are being selected to populate. Consider, for instance, the subdivision of the Val block in Table 2/Figure 3 with reassignment of YUN codons from Val to Leu. In the precursor four-column code (with Val assigned to NUN codons), a cost decrement comes from all of the Val codons in Leu site-types. After reassignment, because of the assumptions on symmetrical (uniform) codon usage, half of the codons in Val site-types code for Leu and half of the codons in Leu site- types code for Val. Because amino acid distance is assumed symmetric, if the frequencies of the Val and Leu site-types were equal, the cost of having 50% Val in Leu stes and 50% Leu in Val sites would be exactly equal to the cost of having 100% Val in Leu sites and 100% Val in Val sites. In fact, if we ignore translational error and the fact that Val codons being reassigned are also filling Ile, Phe and Met and perhaps other site-types (the latter of which at least have quite low frequency), the costs of the old and invading codes would thus be exactly equal, and the change in cost, δΦ, would be exactly zero. So to some large extent, the negative δΦ in the top left of Table 2 comes from the fact that Leu is more abundant than Val in modern-day proteins (in fact, Leu is the most abundant amino acid in the biosphere). I would say that these frequencies, taken from Table 1 of Gilis et al., should be reproduced in this article to emphasize this fact, and that perhaps some playing around with the assumptions on these site- type frequencies might be profitable.

**Author response**: *I agree with the general reasoning that it should be easier to add an amino acid that has a high frequency of sites for which it is optimal. The assumption that the frequency of sites of each type is the same as the frequency of the amino acids in modern proteins is therefore important; however this still seems reasonable to me, and I do not see a better alternative assumption that could be made. The frequencies used are now shown in the final column of Table *1. *It is true that Leu is the most frequent, but this is not the end of the story. Table *2*also shows that it is favourable to add Ile in this position, and Ile has a lower frequency than Val. The tables also show many cases where δΦ is negative, some of which correspond to addition of amino acids with low frequencies*.

For instance, this important role of site-type frequencies raises some questions: first, whether modern-day proteins are a reasonable guide on site requirements of proteins at the time the standard code evolved (were biochemical environments the same? Was the functional repertoire of proteins comparable?), and to what extent amino acid frequencies are determined by codon degeneracies in the genetic code. Recall that King and Jukes [57] used the correlation of amino acid frequencies with codon degeneracy as evidence for their version of the Neutral Theory of Evolution.

Personally, probably like Higgs, I am inclined to believe that codon degeneracies have been shaped in part by relative demand for amino acids in proteins through codon reassignments. In regards to the first question, I think there may be some interesting issues in the evolution of protein requirements over the long term evolution of the biosphere, but I don't find that this raises any serious objections or alternative considerations to Higgs's conclusions – except perhaps in one area. It may be that the discrepancies in Column 4 could be partly explained by, for instance, that Cys and Trp are required more than their frequency in modern-day proteins would suggest. Several have argued that the frequencies of these rare amino acid residues among all proteins in the biosphere are increasing over macroevolutionary time. So, questions like this would seem to beg future investigation.

**Author response**: *Regarding the correlation between amino acid frequencies and codon numbers, I can see some merit in arguing both ways. At first sight, this correlation does not strike me as particularly strong – for example, Leu, with 6 codons, is the most frequent, but Ser and Arg, also with 6, are not particularly frequent (see Table *1). *An interesting comparison would be Leu and Ile, with 6 and 3 codons, respectively. I do not think this is particularly significant and I do not see why these numbers could not have been the other way round. The argument of King and Jukes would be that if substitutions between two amino acids are neutral, then mutation will create more occurrences of the amino acid with the larger number of codons. This would be one reason why Leu is more frequent than Ile. On the other hand, this only works if the amino acids are similar and there is a reasonable chance that a substitution will be neutral. A substitution between Leu and an amino acid like His or Gln in Column 3 is unlikely to be neutral, so the 6:2 ratio of codons for these amino acids does not explain why there is substantially more Leu than either of these. Also, given that there are 2 codons each for His and Gln, codon numbers do not explain why Gln is substantially more frequent than His. Clearly selection on the protein sequence plays a major role in determining amino acid frequencies. Our previous work illustrates this well (Urbina et al*. [18]). *The frequency of amino acids in proteins encoded by mitochondrial genomes varies substantially among species and correlates with the frequency of the nucleotides at fourfold degenerate sites, i.e. biases in mutation rates cause biases in non-synonymous substitutions as well as synonymous ones. The amino acids that vary the most in frequency in response to mutation bias are Ile, Leu and Val, because substitutions among these amino acids are very conservative in physical properties. Gln and His vary much less in frequency among species. We used the physical property distance to predict which amino acids are most variable in response to mutation bias*.

Higgs makes a false distinction between his and our treatments of code-messsage coevolution. He states that our treatment only considers code changes that reduce ambiguity of codons from an initial ambiguous state while his treatment considers reassignments of amino acids on codon blocks. In fact, we treat both codon assignments and codon reassignments in code-message coevolution theory. In fact, we found that the amount and timing of codon reassignments depends strongly on the intensities of mutation and selection on codons [26]. It seems that many in this field find our initially ambiguous codon state to be objectionable. But we show in [26] that – at least with our assumptions on fitness of amino acids within and across sites – our intially ambiguous codon state is very unfit and rapidly selected out of the population either by rapid change of the genetic code away from the ambiguous state, or by purifying selection on genes to reduce the frequency of ambiguous codons. For example, under conditions of weak selection and/or high mutation rates, all initially ambiguous codons are rapidly and redundantly assigned to a small number of amino acids, after which diversifying codon reassignments occur to increase the genetic code vocabulary. In other words, our model produces early genetic codes not unlike the one favored by Higgs! After this initial state, under all parameter ranges we examine, codon reassignments from one amino acid to another can and do occur in our model. The bottom line is that our initially ambiguous codon state is just a symmetrical starting point in our model. And our dynamical conditions are assumed constant over time, so if you object to the initially ambiguous codon state, just concentrate on the latter parts of our code evolution trajectories.

**Author response**: *I have changed the main text to state that the work of Ardell and Sella incorporates both changes from an ambiguous state to a single amino acid and changes from one amino acid to another. The question of whether the initial state was ambiguous is nevertheless an important one. According to the arguments I have given, there could not have been complete ambiguity of all 20 biological amino acids because the later amino acids only arose after the evolution of biochemical pathways. Ambiguous coding for the early amino acids only might have been possible. However, I cannot see why the process of synthesizing random sequences would be selected for, because most of them would be useless and you would never get the same one twice. However, synthesizing specific proteins from a limited repertoire of amino acids seems much more likely to be useful. Furthermore, ambiguous coding would require ambiguous charging of tRNAs. It is not clear to me whether it is simpler to evolve a ribozyme that recognizes a whole range of amino acids and performs the same charging reaction with all of them, or to evolve a ribozyme that recognizes only one amino acid. One might argue that the simpler alternative would be the ancestral state – but is specificity or generality easier to achieve?*

Our conclusion, like Higgs, and like Crick before us, is that the diversification of amino acids in the genetic code is the primary motive force acting on genetic code evolution. Furthermore we agree with Higgs that the standard genetic code's error-minimizing structure is an inevitable consequence of selection to preserve protein-coding information during code evolution. I have a few minor additional comments. I would be most interested to see how robust his results are to the form of the cost (and implicitly, as he points out, fitness) function that he uses eg in Eq. 4. In our work we considered that fitness effects across sites combine multiplicatively, which would seem to me to be better justified than additivity across sites.

**Author response**: *This would be an interesting question for the future. I chose the additive version because this reduces directly to the cost function used by many previous authors in the case where all 20 amino acids are present*.

Higgs's discussion of translational error at the end of his first introductory paragraph considers only misreading and not mischarging.

**Author response**: *It would be interesting to know the relative rates of misreading and mischarging. Presumably a tRNA could be mischarged with any amino acid, not just with one that is assigned to a neighbouring codon. It is not clear that the layout of the code has any effect on the rate of mischarging, so mischarging does not seem so relevant for this paper*.

Many aspects of the discussion seem to me to overemphasize the role of the anticodon in tRNA identity among presumptive primordial aminoacyl-tRNA synthetases. While it is true that the anticodon is an important element for most modern synthetases, there is ample evidence of flexibility, including aminoacylation of acceptor-stem hairpins for about half of the different amino acids. Similarly, because of misacylation error rates, it seems unreasonable to suppose that primordial synthetases would rely on a single base (the second anticodon position) alone to determine identity.

### Reviewer 2

#### Eugene Koonin, National Center for Biotechnology Information, Bethesda, MD

The importance of the code evolution problem cannot be overemphasized. In a sense, this is the central problem in the study of life or, at least, life's evolution (but, "nothing in biology makes sense except in the light of evolution"), the rest is more or less history. The history of the problem itself is quite long, starting with Woese's prescient papers and book of 1965–67. This year we celebrate the 40^th ^anniversary of Crick's 1968 on code evolution. Considering the 40+ years history of the study of code evolution, it is rather shocking to contemplate how little progress has been made as the same questions are addressed today that were the subject of Woese's and Crick's thinking in the 1960ies. Admittedly, the analyses have become much more precise and rigorous. A major part of this methodological tightening is brought about by code cost/fitness functions first introduced by Hurst et al. in the early 1990ies and subsequently modified and elaborated by several researchers. In this paper Higgs extends the cost functions to include codes with fewer than 20 amino acids, a development that provides for exploring the effect of expanding a putative primordial code by recruiting new amino acids. Under a set of reasonable assumptions, the paper shows that, if the code expansion proceeds along a path of minimal disruption of protein structure (maximum robustness to mistranslation), that is, codon sub-series are captured by amino acids similar to the original ones, the code expansion is either outright beneficial or, at worst, very mildly deleterious, hence plausible as an evolutionary scenario. Higgs concludes that "the driving force during this process is not the minimization of translation error, but positive selection for the increased diversity and functionality of the proteins that can be made with a larger amino acid alphabet."

As a general scheme of code evolution, this makes perfect sense. I would only emphasize that this scenario does not render the minimization of translation error "unimportant", but rather incorporates this factor as a constraint that shapes the structure of the code during the expansion of the amino acid alphabet. However, the scenario proposed by Higgs is very specific, with particular amino acids being proposed for each phase of the code evolution. This increases the value of the work but of course, at the same time, makes it more vulnerable to criticism. The scenario starts with a GNN code that encodes only four early amino acids, V, A, D, and G (the plausibility of the appearance of these amino acids on primordial earth is supported by new, apparently very thorough survey by the author himself, and this is of course a strength of the paper), and is rapidly expanded into a 4-column code with the same amino acid alphabet. I am not sure why the transient GNN stage that, as acknowledged by Higgs, does not yield to robust translation, is necessary. It seems to me that the scenario could just as well start directly from the 4-column code, thus avoiding the dubious stage with 48 unassigned codons. Furthermore, it is unclear to me whether or not this scenario has any advantages over the early ideas of Woese and others on the ambiguity of primordial codes whereby multiple codon series initially would collectively – and ambiguously – encode several similar amino acids so that the subsequent evolution of the code would involve (mostly) specialization rather than actual codon reassignment (see Woese [15,16]). It would be interesting to explicitly encode this model using modern approaches and see how it performs.

**Author response**: *These questions are not new, but they are still important. The major advances of this paper over previous treatments are that (i) a more realistic amino acid distance matrix is used, (ii) the cost function is developed so that it can deal with the addition of new amino acids, not just the permutation of the current 20, and (iii) it is possible to make specific predictions about why certain amino acids are assigned to certain positions, rather than generalizations regarding the assignment of similar amino acids to neighbouring codons. As a result of point (ii) it is possible to compare the selective advantage of adding new amino acids and the disadvantage of increasing translational error. This has not been done before in previous treatments of randomized codes. This paper makes important progress in that it proposes evolutionary mechanisms and evolutionary pathways, whereas previous work has focused principally on the statistical comparison of real and random codes*.

*It is possible that the GNN stage was not a necessary predecessor to the four-column stage. However, there must be some reason why G at first position is important. If the four-column code was completely insensitive to the first position base, there is no reason why the four earliest amino acids should have remained assigned to the bottom-row codons*.

*It might be interesting to consider an ambiguous starting condition using a similar approach to that here, but an ambiguous starting condition seems less likely to me, for the reasons stated in response to Reviewer 1*.

More generally, this study shares the essential features of all models of the emergence and early evolution of the code: a reasonable scenario is proposed and carefully explored but there can be no guarantee that this is a necessary scenario. Indeed, as noticed by Higgs, the scenario presented in this paper is compatible with the latest versions of the coevolution hypothesis, and perhaps, with other concepts as well. So are we learning anything substantial about the origin and evolution of the code? I think there is an important message, and this is born out by Higgs's analysis: robustness to translation error is a critically important factor of the code evolution, whether it is considered in terms of direct adaptation or as constraint. Nothing particularly new, the idea was present in Woese's publications over 40 years ago, but I believe this is now established beyond reasonable doubt, and this is certainly worth noting.

**Author response**: *This paragraph rather misrepresents what I have said. The scenario I gave is compatible with some parts of the coevolution theory, but not with others, and the paper clearly explains why I disagree with the other parts. Also the statement that robustness to translational error is critically important misses the point that the predicted pathways of code evolution are more or less the same when the error rate, ε, is zero and when it is non-zero. The pathways are determined by the fact that the new code must not be too disruptive to the function of the genes that evolved under the old code. Translational error is secondary to this*.

Finally, I would note that this careful and interesting study, as well as other studies of the code evolution conducted in the same tradition, by virtue of their design, does not address the central question: how did the coding principle itself evolve? This is a huge problem that can be reasonably approached only in conjunction with the origin of the translation systems, and despite a variety of ideas, there is so far no clear path the putative primordial RNA world to the more modern-like RNA-protein world (see Wolf and Koonin EV [58], and references therein). Once again, no attempt to criticize the present work, just trying to clarify my view of the state of the art in the study of the code evolution.

**Author response**: *Yes, I agree that the stage of code evolution I am addressing is really the closing act of the RNA World, and that a lot has to occur within the RNA World before the translation system can evolve*.

### Reviewer 3

#### Stephen Freeland, University of Maryland, Baltimore County (nominated by Laurence Hurst)

This paper contributes to the extensive literature surrounding the evolution of the standard genetic code. Accepting as a foundation the general observation that codon/amino acid pairings found in the standard genetic code place biochemically similar amino acids next to one another (in terms of mutation or mistranslation), it focuses on the possible evolutionary dynamics by which this pattern could have formed. In particular the author derives a version of the "genetic code coevolution" model according to which new amino acids enter the code by capturing a subset of the codons previously assigned to pre-biotically plausible alternatives. The addition of a new amino acid brings an advantage (of allowing greater protein diversity) and a disadvantage (of disrupting existing protein-coding genes and increasing the scope for mistranslation). By carefully quantifying the biochemical similarity of amino acids, the cost and benefits associated with adding an amino acid to the code, and the prebiotic plausibility of the amino acids the author produces a detailed, quantitative model that provides a vision of how codon assignments of the standard genetic code could have arisen. His major conclusion is that many of the placements of amino acids that we see today could have been associated with an immediate selective advantage to organisms that added them to a primitive code from which they were missing. In general, this is a strong contribution. The author has carefully thought out his model and its assumptions, researched much of the appropriate previous literature and written a mostly clear account of his work.

In a clear and interesting background, referencing of previous work is let down only inasmuch as readers are given no idea just how old and rich the literature is for a GNN primordial genetic code. The observation that GNN codons encode likely primordial amino acids dates back to Crick [14]. It has since formed an explicit start point for many proposals regarding genetic code evolution (e.g. [17,44-47]); likewise, many previous authors have made/discussed the observation that the "columns" of the code (i.e. the amino acids assigned to codons that share a given nucleotide at the second base) show more conserved biochemical features than the "rows" (i.e. the amino acids assigned to codons that share a given nucleotide at the second base) (e.g. [15-17,3]).

**Author response**: *Thankyou for these many useful references. I have added them in the main text*.

Another point is that Wong [59] is often overlooked for having given a first quantitative cost/benefit model for amino acids entering the code.

**Author response**: *Yes, this paper has an interesting discussion of when further additions of amino acids to the code will be favourable, despite the build-up of translational noise. I am confused by this paper, however, because Wong's translational noise decreases as amino acids are added. In my view, translational errors become more important as more amino acids are added and the benefit to adding a new one becomes less because the existing diversity is larger already, so the potential gain in terms of protein function is less. It is the balance of these two things that stops further subdivision of the code after 20 amino acids*.

In the methods sections, the author gives careful consideration to all the concepts on which his quantitative model builds and does a good job of breaking a complex set of metrics into individual steps that, with concentration, are relatively easy to follow. To me it would seem worth explicit mention that equation 2 does not add "weighting" as a new assumption; rather it converts one assumed weighting (equal weight for all dimensions) into another (different weight for all dimensions): this only adds to the authors arguments in favor of dW. Apart from that, my only conceptual concern here relates to the assumption he inherits as to which amino acids are pre-biotically plausible. The author rightly points to a wide general consensus between many different analyses of pre-biotic chemical synthesis (that many have noted, e.g. [41,42]). Specifically, he refers to an (excellent) meta-analysis [23,24] which shows that many, varied estimates of prebiotic plausibility/quantity combine into an index that shows a surprisingly strong correlation with independently made, theoretical thermodynamic calculations for free energy of formation by Amend and Shock [60]. From this both the earlier paper and this one at hand make a key claim that researchers can be confident about what amino acids constitute the "early" set while remaining agnostic about the (controversy surrounding) sources and conditions of prebiotic synthesis. The potential problem I perceive is that although the various experiments and simulations in agreement with one another may seem diverse at first sight, very few have considered high temperature and pressure conditions where (as Higgs and Pudritz note) Amend and Shock's calculations predict very different thermodynamic results. This is relevant because increasing attention is starting to focus on the possibility for a "hot and high pressure" origin for life (this being the exact point of Amend and Shock's paper, and interest value of the differences they predict). While I do not think this is a big problem for the paper at hand, I should like to see some recognition that the confidence placed on "early" amino acids (and thus the GNN model and the rest of the manuscript that follows) all build from an implicit assumption of a cold start to life.

**Author response**: *What is clear from our work is that the observed frequencies in non-biological contexts can be well predicted from the thermodynamic calculations in surface sea-level conditions but not from the calculations at hot, deep-sea conditions. The main controversy around the hydrothermal origin hypothesis is whether molecules formed under such conditions would be stable for any length of time. It may be that Amend and Shock's equilibrium calculations are not really useful for predicting the concentrations of amino acids that would accumulate in hydrothermal systems. Our analysis also incorporated experiments that were intended to represent hydrothermal systems. These also give rise to amino acids similar to those in the Miller-Urey experiments and not to those that are most favourable according to the hydrothermal calculations of Amend and Shock. I do not particularly favour the idea that life began in hydrothermal systems, however, I do not think that our work is a strong argument against this, hence I remain agnostic. The central point of Higgs and Pudritz is that, whatever the calculations say, it is the simplest amino acids that are easiest to form by non-biological chemistry and these are the ones that form in many different observations corresponding to many different physical conditions*.

Moving into the results sections, I encounter the only writing that causes me problems of clarity. We learn that the translational error term is relatively inconsequential when the only differences are between columns (due to the rarity of 2^nd ^position mistranslation: see below), so that the order in which the first amino acids are added has no real effect on Φ. However, "the error term becomes more relevant in later stages of code evolution because the codon blocks become smaller and because the increase in diversity that is gained by addition of the next amino acid becomes less important in comparison with the diversity that is already present in the code." This all makes sense so far, but unless I misunderstand something, when later additions to the code are considered, the mistranslational term presumably picks up increasing cost of 1^st ^position mistranslation, and this will vary with different scenarios for the order and placement of amino acids. Are they added simultaneously? If so, then why? If not, what order is picked and why? Is the effect still not large enough to make a big difference regardless of scenario, or could this term come to dominate the scenarios under which selection creates a code? Shouldn't there be some sort of variance associated with each value in the tables that increases with each table?

**Author response**: *Yes, if ε is non-zero, then the order of addition does make a small difference, and since the error rate at first and third positions is larger, the order of addition of amino acids within a column makes more difference than the order of amino acids in different columns. For each numerical example I gave, I have stated what the prior code was and which new amino acid is being added; therefore there is no confusion. I have considered representative examples, including those which follow the expectations of the theory and those which do not, so I am not picking the order of addition specifically to favour the theory. I am not sure that including more alternative orders would help. I don't know what you mean by variance – variance of what?*

The discussion of problems with "column 4" is interesting but rests heavily on the dogma that second position mistranslation errors are by far the least abundant. This is a view that has come under direct, empirical assault with new and improved techniques for measuring mistranslation *in vivo *(Kramer and Farabaugh [61]). While I do not expect this manuscript to recalculate on this basis, it would be pertinent to remind readers that much of the model presented would require recalculation if the next few years see significant changes to old, weak data regarding mistranslational biases.

**Author response**: *It would certainly be worthwhile having another look if better data on mistranslation rates become available. I would be surprised if the main point that the error rate is smallest at second position does not hold up to future experiments because I think the middle base is the most important for the structure of the codon-anticodon pair. However, I have already shown that the main results are very similar with the current model for errors to the case when there is no error at all (ε = 0.05 and ε = 0 in Tables *2* and *3). *So changing the relative rates of different kinds of errors cannot make a larger difference than this*.

*The central pattern to be explained is that amino acids in the same column are similar, but not those in the same row. Let me state my explanation of this one more time. I showed that evolution favours the addition of amino acids to positions formerly occupied by similar amino acids. I then showed that if the code begins from the simple four-column code in Figure *2, *it will naturally evolve toward a code like the current standard code in which the amino acids in the same column are similar. As a bi-product of this, the effects of translational error will be small compared to randomly reshuffled codes. If the error rates are smallest at second position, this will make the current code appear even more optimized with respect to random codes, because the layout is such that the errors with the biggest effect are those that occur least often. However, the reason that the code has a pattern of similarities by columns is not because the error rate is smallest at second position, it is because the column structure was built in from the beginning and this provided a strong constraint for the subsequent evolution*.

*I will contrast this with the usual argument based on reshuffled codes, which says that the positions of the amino acids were reshuffled in order to minimize the effects of translational error. According to this argument, the asymmetry of the rows and columns arises directly because the error rate at second position is smallest. If the error rates at all positions were equal, then a code with rows and columns exchanged would work equally well, or a code in which rows and columns were similar to an equal extent. Thus, if the relative rates of the errors turn out to be different, this will substantially change the expected outcome according to the reshuffled codes argument, but it will make almost no difference to my argument above*.

Meanwhile broader discussion of the problems that "column 4" seems to present might note that a different interpretation (and mathematical treatment) of stop codons could (I think) produce big changes in the calculations. If mistranslation to and from a stop codon is sufficiently deleterious, then this could presumably absorb much of the negative impact associated with the addition of new amino acids?

**Author response**: *I don't think that stop codons are dealt with very well in any of the literature in this field. The usual assumption is that the three stop codons are fixed in their current position and unable to change. When dealing with randomly reshuffled codes, probably this doesn't matter much. However, for my argument, I do not want to assume that there were stop codons in the current positions from the beginning, because it is more likely that stop codons were a late addition to the code, after the main layout of most of the codons was already established*.

*The cost of mistranslation to/from stop codons does not enter most of the examples given above because there were no stop codons included. Stop codons are included in the final set of examples that follow the pathway proposed by the coevolution theory. Here, the cost of mistranslation of any amino acid to/from a stop is set equal to the largest cost of any amino acid substitution, which is 200 for the Gly-Trp pair in Additional file *1

Another interesting idea to at least mention here would be that amino acids other than the 20 "standard" ones could potentially have entered and then exited the genetic code during its early evolution. In particular, Jukes [62] has argued that Arginine might have been preceded by its biosynthetic precursor Ornithine. This brings up the much neglected topic of why these particular 20 amino acids were chosen from the hundreds or thousands that were available. It would be hard to include "non-coded" amino acids into this model, but it would not hurt to explain why this is so and to discuss the relevance of thinking about this.

**Author response**: *It seems speculative to propose that other amino acids were added and then subsequently removed, when we have no evidence for this. It is interesting to consider why the other amino acids did not end up in the code. I am afraid the theory given here does not have much to say about this*.

All in all this is a novel and worthwhile addition to the genetic code literature. As the author points out in the introduction, relatively little of the genetic code literature has made quantitative models/predictions about the pathways by which the standard code emerged. There are some interesting ideas here, and a lot of room for others to tweak and test variations of the model and its associated predictions so as to produce (over time) a broad context for thinking about the processes described here.

## Supplementary Material

Additional file 1 **Table S1**. Weighted Property distance matrix d_*W*_, scaled such that the mean is 100 and rounded to nearest integer. Click here for file

## References

[B1] Haig D, Hurst LD (1991). A quantitative measure of error minimization in the genetic code. J Mol Evol.

[B2] Freeland SJ, Hurst LD (1998). The genetic code is one in a million. J Mol Evol.

[B3] Freeland SJ, Hurst LD (1998). Load minimisation of the code: history does not explain the pattern. Proc Roy Soc Lond B.

[B4] Freeland SJ, Knight RD, Landweber LF, Hurst LD (2000). Early fixation of an optimal code. Mol Biol Evol.

[B5] Freeland SJ, Wu T, Keulmann N (2003). The case for an error minimizing standard genetic code. Orig Life Evol Biosph.

[B6] Gilis D, Massar S, Cerf NJ, Rooman M (2001). Optimality of the genetic code with respect to protein stability and amino acid frequencies. Genome Biology.

[B7] Goodarzi H, Nejad HA, Torabi N (2004). On the optimality of the genetic code with the consideration of termination codons. BioSystems.

[B8] Goodarzi H, Najafabadi HS, Hassani K, Nejad HA, Torabi N (2005). On the optimization of the genetic code with the consideration of coevolution theory by comparison of prominent cost measure matrices. J Theor Biol.

[B9] Knight RD, Freeland SJ, Landweber LF (2001). Rewiring the keyboard: Evolvability of the genetic code. Nature Reviews Genetics.

[B10] Sengupta S, Higgs PG (2005). A unified model of codon reassignment in alternative genetic codes. Genetics.

[B11] Sengupta S, Yang X, Higgs PG (2007). The mechanisms of codon reassignments in mitochondrial genetic codes. J Mol Evol.

[B12] Di Giulio M, Medugno M (2001). The level and landscape of optimization in the origin of the genetic code. J Mol Evol.

[B13] Novozhilov AS, Wolf YI, Koonin EV (2007). Evolution of the genetic code: partial optimization of a random code for robustness to translational error in a rugged fitness landscape. Biology Direct.

[B14] Crick FHC (1968). The origin of the genetic code. J Mol Biol.

[B15] Woese CR (1965). Order in the genetic code. Proc Natl Acad Sci USA.

[B16] Woese CR (1967). On the evolution of the genetic code. Proc Natl Acad Sci USA.

[B17] Taylor FJ, Coates D (1989). The Code Within the Codons. Bio Systems.

[B18] Urbina D, Tang B, Higgs PG (2006). The response of amino acid frequencies to directional mutation pressure in mitochondrial genome sequences is related to the physical properties of the amino acids and to the structure of the genetic code. J Mol Evol.

[B19] Wong JT (1975). A co-evolution theory of the genetic code. Proc Nat Acad Sci USA.

[B20] Wong JT (2005). Coevolution theory of the genetic code at age thirty. BioEssays.

[B21] Di Giulio M (2005). The origin of the genetic code: theories and their relationships, a review. BioSystems.

[B22] Di Giulio M (2008). An extension of the coevolution theory of the origin of the genetic code. Biology Direct.

[B23] Higgs PG, Pudritz RE, Pudritz RE, Higgs PG, Stone J (2007). From protoplanetary disks to prebiotic amino acids and the origin of the genetic code. Planetary systems and the origins of life.

[B24] Higgs PG, Pudritz RE (2009). A thermodynamic basis forprebiotic amino acid synthesis and the nature of the first geneticcode. Astrobiology.

[B25] Ardell DH, Sella G (2001). On the evolution of redundancy in genetic codes. J Mol Evol.

[B26] Ardell DH, Sella G (2002). No accident: genetic codes freeze in error-correcting patterns of the standard genetic code. Phil Trans R Soc Lond B.

[B27] Sella G, Ardell DH (2002). The impact of message mutation on the fitness of a genetic code. J Mol Evol.

[B28] Sella G, Ardell DH (2006). The coevolution of genes and genetic codes: Crick's frozen accident revisited. J Mol Evol.

[B29] Vetsigian K, Woese C, Goldenfeld N (2006). Collective evolution and the genetic code. Proc Nat Acad Sci USA.

[B30] Woese CR, Dugre DF, Dugre SA, Kondo M, Saxinger WC (1966). On the fundamental nature and evolution of the genetic code. Cold Spring Harbor Symp Quant Biol.

[B31] Higgs PG, Attwood TK (2005). Bioinformatics and Molecular Evolution.

[B32] Creighton TE (1993). Proteins: structures and molecular properties.

[B33] Zimmerman JM, Eliezer N, Simha R (1968). The characterization of amino acid sequences in proteins by statistical methods. J Theor Biol.

[B34] Kyte J, Doolittle RF (1982). A simple method for displaying the hydropathic character of a protein. J Mol Biol.

[B35] Engelman DA, Steitz TA, Goldman A (1986). Identifying non-polar transbilayer helices in amino acid sequences of membrane proteins. Annu Rev Biophys Biophys Chem.

[B36] Miller S, Janin J, Lesk AM, Chothia C (1987). Interior and surface of monomeric proteins. J Mol Biol.

[B37] Rose GD, Geselowitz AR, Lesser GJ, Lee RH, Zehfus MH (1985). Hydrophobicity of amino acid residues in globular proteins. Science.

[B38] Higgs PG, Hao W, Golding GB (2007). Identification of selective effects on highly expressed genes. Evol Bioinform Online.

[B39] Waglechner N (2008). Protein evolution in microbial extremophiles. MSc Thesis.

[B40] Heady RB, Lucas JL Permap 11.6e – Perceptualmapping using interactive multidimensional scaling. http://www.ucs.louisiana.edu/~rbh8900/permap.html.

[B41] Wong JT, Bronskill PM (1979). Inadequacy of pre-biotic synthesis as the origin of proteinaceous amino acids. J Mol Evol.

[B42] Weber AL, Miller SL (1981). Reasons for the occurrence of the twenty coded protein amino acids. J Mol Evol.

[B43] Trifonov EN (2000). Consensus temporal order of amino acids and the evolution of the triplet code. Gene.

[B44] Dillon LS (1973). The origins of the genetic code. Botanical Rev.

[B45] Miseta A (1989). The role of protein associated amino acid precursor molecules in the organisation of genetic codons. Physiol Chem Phys Med NMR.

[B46] Hartman H (1995). Speculations on the origin of the genetic code. J Mol Evol.

[B47] Brooks DJ, Fresco JR (2003). Greater GNN pattern bias in sequence elements encoding conserved residues of ancient proteins may be an indicator of amino acid composition of early proteins. Gene.

[B48] Trifonov EN (1987). Translation framing code and frame-monitoring mechanism as suggested by the analysis of mRNA and 16S rRNA sequences. J Mol Biol.

[B49] Mark C, Grosjean H (2002). tRNomics: Analysis of tRNA genes from 50 genomes of Eukarya, Archaea and Bacteria reveals anticodon-sparing strategies and domain-specific features. RNA.

[B50] Tong KL, Wong JT (2004). Anticodon and wobble evolution. Gene.

[B51] Higgs PG (2000). RNA Secondary Structure: Physical and Computational Aspects. Quart Rev Biophys.

[B52] Osawa S, Jukes TH (1989). Codon reassignment (codon capture) in evolution. J Mol Evol.

[B53] Knight RD, Landweber LF (2000). Guilt by association: the arginine case revisited. RNA.

[B54] Caporaso JG, Yarus M, Knight R (2005). Error minimization and coding triplet/binding site associations are independent features of the canonical genetic code. J Mol Evol.

[B55] Yarus M, Caporaso JG, Knight R (2005). Origins of the genetic code: the escaped triplet theory. Annu Rev Biochem.

[B56] Di Giulio M, Medugno M (1999). Physicochemical optimization in the genetic code origin as the number of codified amino acids increases. J Mol Evol.

[B57] King JL, Jukes TH (1969). Non-Darwinian Evolution. Science.

[B58] Wolf YI, Koonin EV (2007). On the origin of the translation system and the genetic code in the RNA world by means of natural selection, exaptation, and subfunctionalization. Biol Direct.

[B59] Wong JT (1976). The evolution of a universal genetic code. Proc Natl Acad Sci USA.

[B60] Amend JP, Shock EL (1998). Energetics of Amino Acid Synthesis in Hydrothermal Ecosystems. Science.

[B61] Kramer EB, Farabaugh PJ (2007). The frequency of translational misreading errors in E. coli is largely determined by tRNA competition. RNA.

[B62] Jukes TH (1973). Arginine as an evolutionary intruder into protein synthesis. Biochem Biophys Res Commun.

