# Supplementary material for: A four-column theory for the origin of the genetic code: tracing the evolutionary pathways that gave rise to an optimized code
Source: Biol Direct. 2009 Apr 24;4:16. doi: 10.1186/1745-6150-4-16 (PMC2689856; doi:10.1186/1745-6150-4-16)
Supplement: Additional file 1 — Table S1. Weighted Property distance matrix dW, scaled such that the mean is 100 and rounded to nearest integer. [file 1745-6150-4-16-S1.doc]

Table S1 - Weighted Property distance matrix d_W_, scaled such that the mean is 100 and rounded to nearest integer.

F L I M V S P T A Y H Q N K D E C W R G

F 0 38 39 30 56 134 111 97 117 61 98 120 137 146 160 144 81 59 131 172

L 38 0 12 46 20 123 98 85 98 82 107 124 135 152 158 148 66 90 147 159

I 39 12 0 50 22 131 109 95 104 90 115 134 145 161 167 157 68 94 155 165

M 30 46 50 0 59 108 91 75 94 52 76 99 114 130 139 126 63 69 116 145

V 56 20 22 59 0 119 98 84 90 96 113 129 136 158 156 149 61 107 156 152

S 134 123 131 108 119 0 57 48 41 116 84 77 57 112 80 91 91 157 126 54

P 111 98 109 91 98 57 0 23 68 85 71 56 58 89 89 86 96 123 105 108

T 97 85 95 75 84 48 23 0 51 79 62 60 60 98 90 88 75 116 107 97

A 117 98 104 94 90 41 68 51 0 119 97 102 88 136 107 116 61 153 146 64

Y 61 82 90 52 96 116 85 79 119 0 60 73 100 98 129 107 105 44 80 162

H 98 107 115 76 113 84 71 62 97 60 0 47 59 79 87 72 101 93 64 124

Q 120 124 134 99 129 77 56 60 102 73 47 0 35 49 66 48 124 112 61 126

N 137 135 145 114 136 57 58 60 88 100 59 35 0 68 40 40 122 139 86 100

K 146 152 161 130 158 112 89 98 136 98 79 49 68 0 89 67 163 130 46 158

D 160 158 167 139 156 80 89 90 107 129 87 66 40 89 0 32 144 163 110 111

E 144 148 157 126 149 91 86 88 116 107 72 48 40 67 32 0 144 140 85 131

C 81 66 68 63 61 91 96 75 61 105 101 124 122 163 144 144 0 126 157 109

W 59 90 94 69 107 157 123 116 153 44 93 112 139 130 163 140 126 0 104 200

R 131 147 155 116 156 126 105 107 146 80 64 61 86 46 110 85 157 104 0 169

G 172 159 165 145 152 54 108 97 64 162 124 126 100 158 111 131 109 200 169 0
